# Single-cell RNA sequencing reveals the transcriptomic characteristics of peripheral blood mononuclear cells in hepatitis B vaccine non-responders

**DOI:** 10.3389/fimmu.2023.1091237

**Published:** 2023-08-01

**Authors:** Meie Zhao, Chunxia Wang, Peiqiang Li, Tao Sun, Jing Wang, Shasha Zhang, Qinglong Ma, Fengdie Ma, Wenjing Shi, Maoning Shi, Yapeng Ma, Yunyan Pan, Hui Zhang, Xiaodong Xie

**Affiliations:** ^1^ School of Basic Medical Sciences, Lanzhou University, Lanzhou, Gansu, China; ^2^ Department of Laboratory Medicine, The First People’s Hospital of Lanzhou, Lanzhou, Gansu, China; ^3^ Clinical Laboratory, Huzhou Central Hospital, Huzhou Hospital Affiliated with Zhejiang University, Huzhou, Zhejiang, China; ^4^ Department of Laboratory Medicine, The First People’s Hospital of Tianshui, Tian Shui, Gansu, China; ^5^ Department of Laboratory Medicine, The Second Hospital of Lanzhou University, Lanzhou, Gansu, China; ^6^ Virus Laboratory, Gansu Provincial Center for Disease Control and Prevention, Lanzhou, Gansu, China

**Keywords:** hepatitis B vaccine, no immune response, high immune response, peripheral blood mononuclear cell, single-cell RNA sequencing

## Abstract

The emergence of a vaccine against hepatitis B has proven to be an important milestone in the prevention of this disease; however, 5%–10% of vaccinated individuals do not generate an immune response to the vaccine, and its molecular mechanism has not been clarified. In this study, single-cell RNA sequencing was performed on peripheral blood mononuclear cells (PBMCs) from three volunteers with a high immune response (HR) and three with no immune response (NR) to the hepatitis B vaccine. We found that the antigen-presenting activity scores of various antigen-presenting cells, the mitogen-activated protein kinase (MAPK) pathway activity scores of naive B cells, and the cell activity scores of three types of effector T cells were significantly decreased, whereas the cytotoxicity scores of CD3^high^CD16^low^KLRG1^high^ natural killer T (NKT) cells were significantly increased in the NR group compared with those in the HR group. Additionally, the expression levels of some classical molecules associated with distinct signaling pathways—including *HLA-B*, *HLA-DRB5*, *BLNK*, *BLK*, *IL4R*, *SCIMP*, *JUN*, *CEBPB*, *NDFIP1*, and *TXNIP*—were significantly reduced in corresponding subsets of PBMCs from the NR group relative to those of the HR group. Furthermore, the expression of several cytotoxicity-related effector molecules, such as *GNLY*, *NKG7*, *GZMB*, *GZMM*, *KLRC1*, *KLRD1*, *PRF1*, *CST7*, and *CTSW*, was significantly higher in CD3^high^CD16^low^KLRG1^high^ NKT cells derived from non-responders. Our study provides a molecular basis for the lack of response to the hepatitis B vaccine, including defective antigen presentation, decreased T cell activity, and reduced IL-4 secretion, as well as novel insight into the role of NKT cells in the immune response to the hepatitis B vaccine.

## 1 Introduction

The emergence of the hepatitis B vaccine is an important milestone in the prevention of hepatitis B; however, approximately 5%–10% (1) of individuals do not produce an immune response to the existing recombinant hepatitis B vaccine (2, 3). The reason for the lack of an immune response and the characteristics of peripheral blood mononuclear cells (PBMCs) in non-responders are still not fully understood.

To date, studies investigating the absence of an immune response to the hepatitis B vaccine have mainly been carried out at the population level. However, the heterogeneity among cells that play an important role in regulating cellular function is often masked by the population signal (4). Single-cell RNA sequencing (Single-cell RNA-seq) technology has been applied in the field of immunology and revealed the heterogeneity among immune cells (5). In this study, using this technology, we generated a comprehensive transcriptomic landscape of PBMCs from non-responders who received enhanced immunization with recombinant hepatitis B vaccine and identified several key cell type-specific molecules that may serve as targets for enhancing the immune response to the hepatitis B vaccine.

## 2 Subjects and methods

### 2.1 Subjects

From January 2020 to March 2021, a total of 36 volunteers were recruited from the first People’s Hospital of Lanzhou, Gansu Province, including six who did not develop an immune response (NR) and 30 who displayed a high immune response (HR) to the recombinant hepatitis B vaccine. All the volunteers met all the following criteria: (1) at least 18 years of age, standard body mass index and transaminase, and no current or past history of the disease; (2) negative for serum markers of hepatitis B and had a history of hepatitis B vaccination (all serum markers are described in **Supplementary Methods**); (3) willing to be inoculated with 20 μg/ml recombinant hepatitis B vaccine (Shenzhen Kangtai Biological Products Co., Ltd, Shenzhen, China) following a 0-, 1-, and 6-month schedule. One month after the third dose of the hepatitis B vaccine, participants whose serum concentrations of the hepatitis B surface antibody (HBsAb) were lower than 10 mIU/ml were considered non-responders and those whose HBsAb titer was greater than or equal to 1,000 mIU/ml were considered high responders (HR). The instruments and reagents used for the detection of serum markers are provided in **Supplementary Methods**.

### 2.2 Sample collection and PBMC isolation

One month after the administration of the third dose of the vaccine, 8 ml of peripheral venous blood was collected from each volunteer. Whole blood was diluted with an equal volume of phosphate-buffered saline (PBS) (Tianjin Hao Yang Biological Manufacture Co., Ltd, Tianjin, China), transferred to a centrifuge tube containing Human Lymphocyte Separation Medium (Dakewe Biotech Co., Ltd, Shenzhen, China), and centrifuged at 700×*g* for 20 min. PBMCs, located between the plasma layer and the cell separation medium, were carefully transferred into another centrifuge tube and washed twice with PBS.

### 2.3 Preparation of single-cell suspensions

PBMCs obtained from three high responders and three non-responders matched for age, sex, and ethnicity were used for single-cell RNA-seq. The PBMCs were resuspended in 1 ml of Iscove’s modified Dulbecco’s medium supplemented with 10% fetal bovine serum and a single-cell suspension was prepared. Cell counts and cell survival (greater than 85%) were determined using a Luna-FL automatic cell counter. Dead cells were removed using a dead cell removal kit. Finally, the cell concentration was adjusted to 700–1,200 cells/μl.

### 2.4 Single-cell RNA-seq

Single-cell RNA-seq libraries were constructed using a Single Cell 3′ Library and Gel Bead Kit (v3.1; 10X Genomics, 1000121) and a Chromium Single Cell A Chip Kit (10X Genomics, 1000120) according to the manufacturer’s instructions. Single cells were suspended in PBS containing 0.04% BSA (700–1,200 living cells per ml as determined using the LUNA-FL cell counter) and loaded onto a Chromium single-cell controller (10X Genomics) to generate single-cell gel bead-in-emulsion (GEMs) according to the manufacturer’s protocol. Approximately 10,000 cells were added to each channel and approximately 10,000 target cells were captured from each sample. The captured cells were lysed, and the released RNA was barcoded through reverse transcription in each GEM. Reverse transcription was performed using a C1000 Touch Thermal Cycler (Bio-Rad) at 53°C for 45 min, followed by 85°C for 5 min, and held at 4°C. The resulting cDNA was amplified and then assessed for quality using an Agilent 4200 TapeStation. The libraries were paired-end (150 bp) sequenced using the Illumina NovaSeq 6000 system. The above experiments were carried out by BioMiao Biological Technology Co., Ltd, Beijing, China.

### 2.5 Single-cell RNA-seq data processing

Raw gene expression matrices were generated for each sample using the Cell Ranger (v.4.0.0) pipeline coupled with the human transcriptome reference version GRCh38-2020-A. The generated filtered gene expression matrices were analyzed by R software (v.4.1.1) with the Seurat 4 package (v.4.0.0). In brief, genes that were expressed in more than 1% of cells were discarded, while cells were selected for further analyses if they met the following criteria: (1) 500 < unique molecular identifiers (UMIs) < mean + 2 standard deviations; (2) 400<genes<mean + 2 standard deviations; (3) <10% mitochondrial genome. The gene expression matrices were then normalized using LogNormalize in Seurat and 2,000 features with high cell-to-cell variation were calculated using the FindVariableFeatures function. To reduce the dimensionality of the datasets, the RunPCA function was run with default parameters on linear-transformation scaled data generated by the ScaleData function. Next, the true dimensionality of each dataset was identified using the functions ElbowPlot, DimHeatmap, and JackStrawPlot according to the recommendations of the Seurat developers. Finally, the top 50 principal components were clustered using the FindNeighbors and FindClusters functions, and non-linear dimensional reduction was performed using the RunUMAP function with default settings. All the details of the Seurat analyses that are relevant to this study can be found in the website tutorial (https://satijalab.org/seurat/articles/pbmc3k_tutorial.html).

### 2.6 Integration of different datasets

To compare cell types and the proportions of each cell type between the NR group and the HR group, six datasets were integrated using canonical correlation analysis plus mutual nearest neighbor (CCA+MNN) (6). Finally, six distinct single-cell RNA-seq datasets were assembled into an integrated and unbatched dataset.

### 2.7 Identification of cell type

The FindAllMarkers function was used to find markers for each cluster. Clusters were then classified and annotated based on the expression of canonical markers. Clusters that expressed two or more canonical cell-type markers were classified as doublet cells and excluded from further analysis.

### 2.8 Identification and functional enrichment analysis of differentially expressed genes

The FindMarkers function and the Wilcox rank-sum test in Seurat were used to analyze gene expression levels in each cell cluster. Finally, DEGs were screened according to the following conditions: (1) Avg_log_2_ (fold change) > 0.25; (2) min. PCT > 0.1; and (3) *P*. value _ adjust < 0.05. Functional enrichment analysis of the DEGs was conducted using the clusterProfiler package (7), Metascape (8), and the STRING database (9).

### 2.9 Expression enrichment scores of cell-state genes

The UCELL package (10) was used to calculate the scores of the expression of HLA-related, cytotoxic activity-related, MAPK pathway-related, and cell activity-related gene sets. According to GSEA (Molecular Signature Database) and related literature (11–15), we selected 10 cytotoxicity-related genes, *NKG7*, *CCL4*, *CST7*, *PRF1*, *GZMA*, *GNLY*, *GZMH*, *KLRB1*, *KLRD1*, and *CTSW*, and 17 MAPK pathway-related genes, *DDIT4*, *DUSP1*, *FOS*, *FOSB*, *JUN*, *JUNB*, *JUND*, *DDX5*, *PDCD4*, *DNAJA1*, *BLK*, *BLNK*, *UBA52*, *CXCR4*, *HSPA8*, *NFKBIA*, and *NFKBID*. The cell activity-related genes were derived from the Gene Ontology (GO) Biological Process category and comprised *ACTB*, *ACTG1*, *ANXA1*, *RHOA*, *B2M*, *ZFP36L2*, *CD8B*, *CD44*, *KLF6*, *FCGR3A*, *GNAS*, *HLA-A*, *HLA-E*, *HMGB1*, *IL7R*, *ITGB1*, *ITGB2*, *JUN*, *MSN*, *MYH9*, *PRKCB*, *PTGDS*, *RPS6*, *CCL3*, *VAMP2*, *TLN1*, *YY1*, *ITM2A*, *KLRK1*, *KMT2E*, *DOCK8*, *JAML*, and *NCR3*.

### 2.10 RT-qPCR

Total RNA was extracted from PBMCs using a GeneJET RNA Purification Kit (Thermo Fisher Scientific, USA) according to the manufacturer’s instructions. RNA concentration (ng/μl) and purity (260/280 ratio) were determined using a NanoDrop One UV-Vis Spectrophotometer (Thermo Fisher Scientific, USA). The β-actin gene served as the internal control. All primers were designed by Primer-Blast and spanned exon–exon junctions. Extracted RNA was reverse transcribed to cDNA using a Goldenstar RT6 cDNA Synthesis Kit Ver.2 (Tsingke Biotechnology Co., Ltd, Beijing, China). qPCR was performed using TSINGKE Master SYBR Green I qPCR Mix (Tsingke Biotechnology Co., Ltd, Beijing, China) with a QuantStudio™ 5 Real-Time PCR System according to the manufacturer’s instructions. Relative gene expression levels were calculated using the 2^−ΔΔCt^ method. The qPCR results were plotted and analyzed in GraphPad Prism v7.0 and are expressed as mean ± standard deviation. The Mann–Whitney U Test was used to compare differences between independent groups and a *P*-value <0.05 was considered significant. The sequences of the primers used for qPCR were (5′–3′) β-actin, forward 5′-CCTTCCTGGGCATGGAGTC-3′ and reverse 5′-TGATCTTCATTGTGCTGGGTG-3′; HLA-B, forward 5′-AGCCATCTTCCCAGTCCA-3′ and reverse 5′-AGCTCCGATGACCACAA-3′; TXNIP, forward 5′-CCAGCACTTGGTCAGTCA-3′ and reverse 5′-CCATCTTCAGCCCACACT-3′; and JUN, forward 5′-GAGCATGACCCTGAACCTG-3′ and reverse 5′-CCGTTGCTGGACTGGATT-3′. Details of the RT-qPCR assay are described in **Supplementary Methods**.

## 3 Results

### 3.1 Single-cell atlas of PBMCs from non-responders and high responders

**Figure 1:**
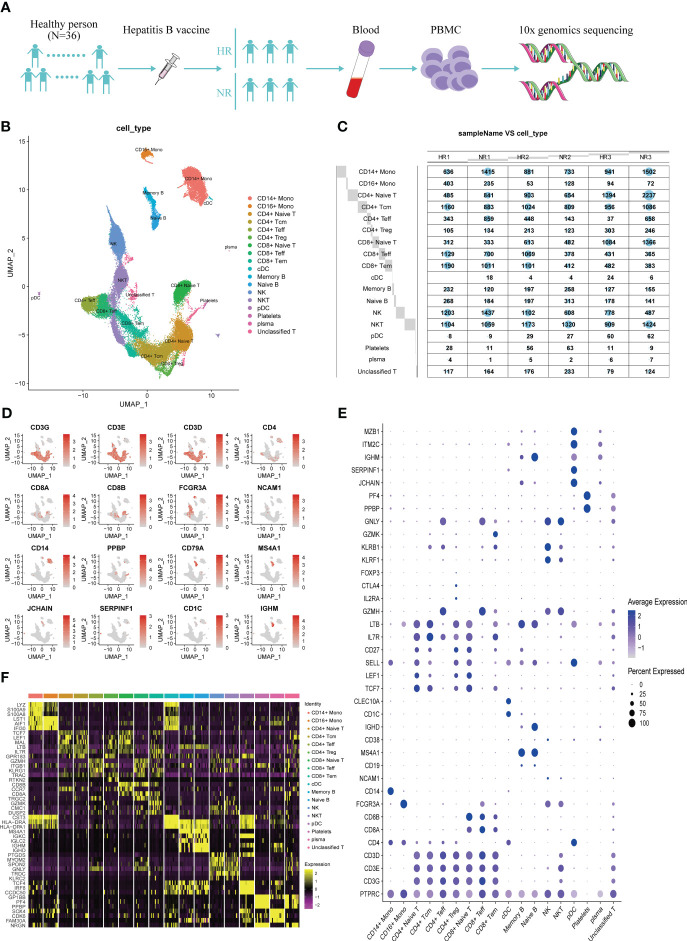
The transcriptional map of peripheral blood mononuclear cells (PBMCs) from non-responders (NR) and high responders (HR) to the hepatitis B vaccine. **(A)** Flowchart showing the overall study design; 3′ single-cell RNA sequencing was undertaken for PBMCs from three NR and three HR volunteers. **(B)** A UMAP showing the 18 cell clusters identified from 52,299 high-quality cells derived from both groups; each dot represents one cell and dots are colored according to the cell type. **(C)** A balloon plot showing the number of cells that were captured from the various cell types in each sample. **(D)** A UMAP plot displaying the classical marker genes used to identify each cell type; genes are colored according to their expression level in the cells. **(E)** A dot plot showing the expression level of the selected classical marker genes in the 18 cell clusters; the shade of the dot represents the expression level, and the size of the dot represents the proportion of cells expressing the gene. **(F)** A heat map showing the three most highly expressed genes in each of the 18 cell clusters.

To characterize the immunological characteristics of non-responders and high responders to the hepatitis B vaccine, we performed single-cell RNA-seq on PBMCs from the three volunteers in each group (**Figure 1A**). The characteristics of the six samples are detailed in **Supplementary Table 1**. After quality control of the sequencing data, 52,299 high-quality cells were obtained, including 26,434 from the three non-responders and 25,865 from the three high responders. All these cells were grouped in 28 clusters according to the transcription characteristics of the top 50 principal components (**Supplementary Figures 1A–F**). Based on the expression of canonical marker genes, we identified 17 major cell types among the 28 clusters (**Figures 1B–F**; **Supplementary Table 2**). No difference in the percentage of each cell type was found between the two groups (*P*>0.05) (**Supplementary Figure 1G**).

### 3.2 Transcriptional characteristics of antigen-presenting cells from non-responders

**Figure 2:**
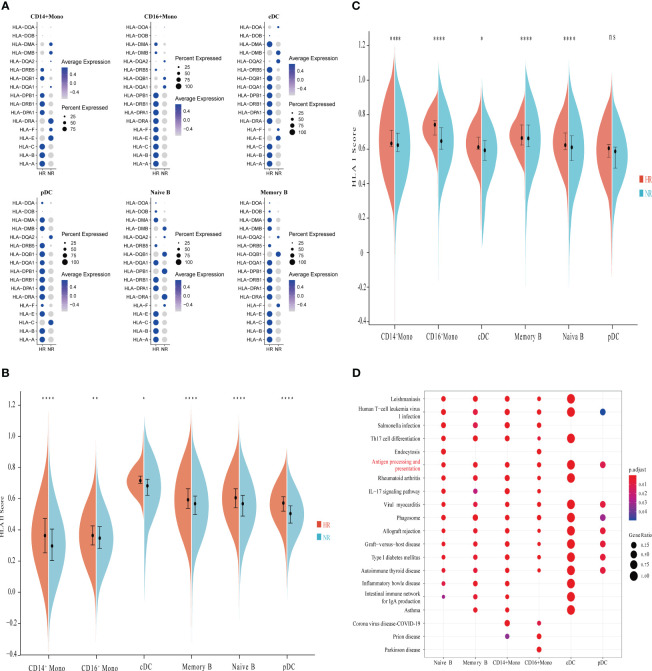
Comparison of the characteristics of antigen-presenting cells (APCs) between the non-responder (NR) and high-responder (HR) groups. **(A)** A dot plot showing the proportions of cells expressing HLA-related genes and the average expression levels of HLA-related genes in APCs from the NR and HR groups. **(B, C)** A violin diagram showing the expression scores of HLA-I- and HLA-II-related genes in APCs from the NR and HR groups; the horizontal line dividing the box represents the median value and the two ends of the box represent the upper quartile (Q3) and lower quartile (Q1); the difference between Q1 and Q3 is the interquartile range (IQR). A *P*-value <0.05 was considered significant. *P<0.05; **P<0.01; ****P<0.0001; ns, not significant (Student’s *t*-test). B indicates the expression score of HLA-II-related genes and C indicates the expression score of HLA-I-related genes. **(D)** Enrichment analysis of genes with low expression in APCs; the Kyoto Encyclopedia of Genes and Genomes (KEGG) terms of interest are shown in red.

As previously shown in **Figure 1**, six types of APCs were identified, including naive B cells, memory B cells, CD14^+^ monocytes, CD16^+^ monocytes, classical dendritic cells (cDCs), and plasmacytoid dendritic cells (pDCs). We first examined the expression of HLA-I and HLA-II molecules in the APCs and found significant heterogeneity in the expression of these molecules between the APCs of the two groups (**Figure 2A**). Differential analysis indicated that, in the NR group, the expression level of HLA-DRB5 was markedly downregulated in several APCs, while HLA-B was significantly underexpressed in CD14^+^ monocytes, CD16^+^ monocytes, and pDCs relative to the HR group (**Supplementary Figure 2**; **Supplementary Table 3**).

We further found that the expression score for HLA-II molecules in the various APCs was significantly lower in the NR group than in the HR group (**Figure 2B**). Except for pDCs, the expression score of HLA-I molecules in the APCs was also significantly lower in non-responders than in high responders (**Figure 2C**). Kyoto Encyclopedia of Genes and Genomes (KEGG) pathway enrichment analysis showed that the downregulated genes in the various APCs of the NR group were significantly enriched in the antigen processing and presentation pathway (**Figure 2D**).

### 3.3 Features of humoral immunity in non-responders

**Figure 3:**
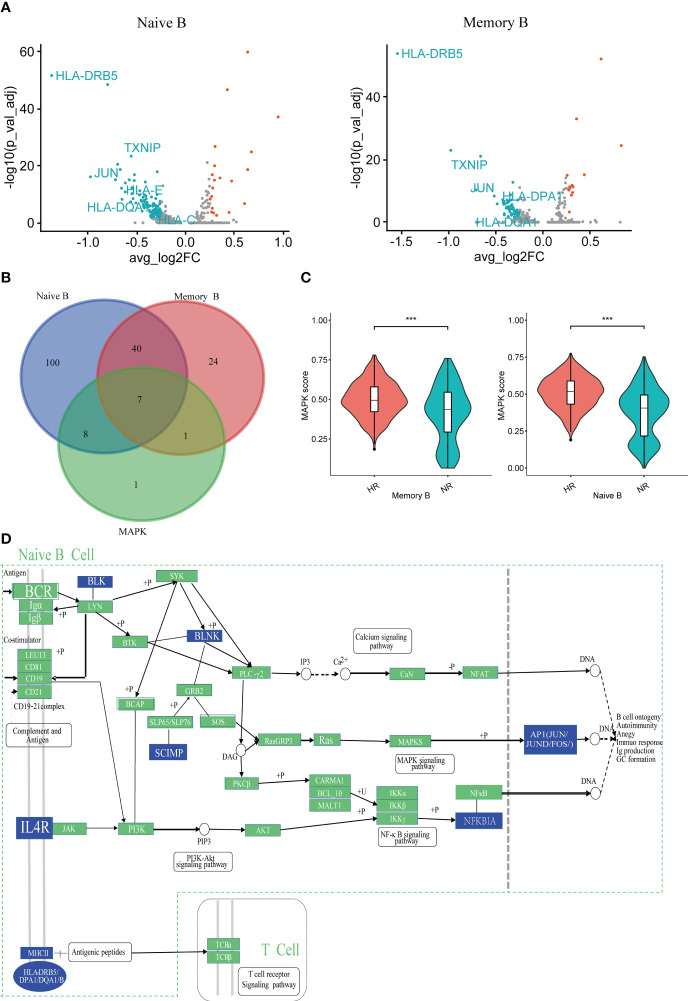
The characteristics of humoral immunity in the non-responder (NR) and high-responder (HR) groups. **(A)** A volcano plot showing the differentially expressed genes (DEGs) as determined by the Wilcox rank-sum test (NR group *vs*. the HR group); adjusted *P*-values <0.05 and |avg_log2FC| > 0.25 indicate that the difference is significant. The blue dots and red dots, respectively, represent downregulated and upregulated genes in naive B cell or memory B cell subsets from the NR group; the genes of interest are marked with the respective symbol. **(B)** A Venn diagram showing the overlap between MAPK-related genes and the DEGs of two B cell subsets from the NR group. **(C)** A violin diagram showing the expression scores of MAPK-related DEGs in memory B cells and naive B cells from the NR group. A *P*-value <0.05 was considered significant. ***P<0.001 (Student’s *t*-test). **(D)** A Kyoto Encyclopedia of Genes and Genomes (KEGG) network showing the regulatory information of some downregulated genes in naive B cells from the NR group; the blue squares represent downregulated genes.

To screen for the key cells and molecules that regulate the humoral immune response, we analyzed the DEGs in the naive B cell and memory B cell subsets between the NR and HR groups. A total of 155 DEGs were identified in the naive B cell subset, accounting for 3.95% (155/3,925) of the total number of captured genes; this number was higher than that detected in the memory B cell subset, in which 72 DEGs were identified, accounting for 2.17% (72/3479) of the total number of captured genes (**Figure 3A**; **Supplementary Table 4**). Additionally, in the protein–protein interaction (PPI) network constructed with the DEGs, the average node degree and the number of nodes were higher, and the edges were more numerous, in the naive B cell subset than in the memory B cell subset (**Supplementary Table 5**). Meanwhile, 15 MAPK pathway-related DEGs were identified in the naive B cell subset, 14 of which were downregulated and one upregulated in the NR group relative to that in the HR group; eight DEGs were identified in the memory B cell subset, including seven that were downregulated and one that was upregulated in the NR group (**Figure 3B**; **Supplementary Figures 3A, B**; **Supplementary Table 6**). Furthermore, the MAPK pathway scores of the naive B cell and memory B cell subsets were lower in the NR group than in the HR group, with the lowest score being observed in the NR-derived naive B cell subset (**Figure 3C**; **Supplementary Figure 3C**). Additionally, we found that several molecules (*BLNK*, *BLK*, *IL4R*, *SCIMP*, *NFKBIA*, *JUN*, and *JUND*, among others) that act downstream of the B cell and IL4R receptor signaling pathways and participate in Ca^2+^, MAPK, and NF-κB signaling were downregulated in the naive B cell subset in the NR group (**Figure 3D**; **Supplementary Figure 3D**).

### 3.4 Characteristics of adaptive immunity in non-responders

**Figure 4:**
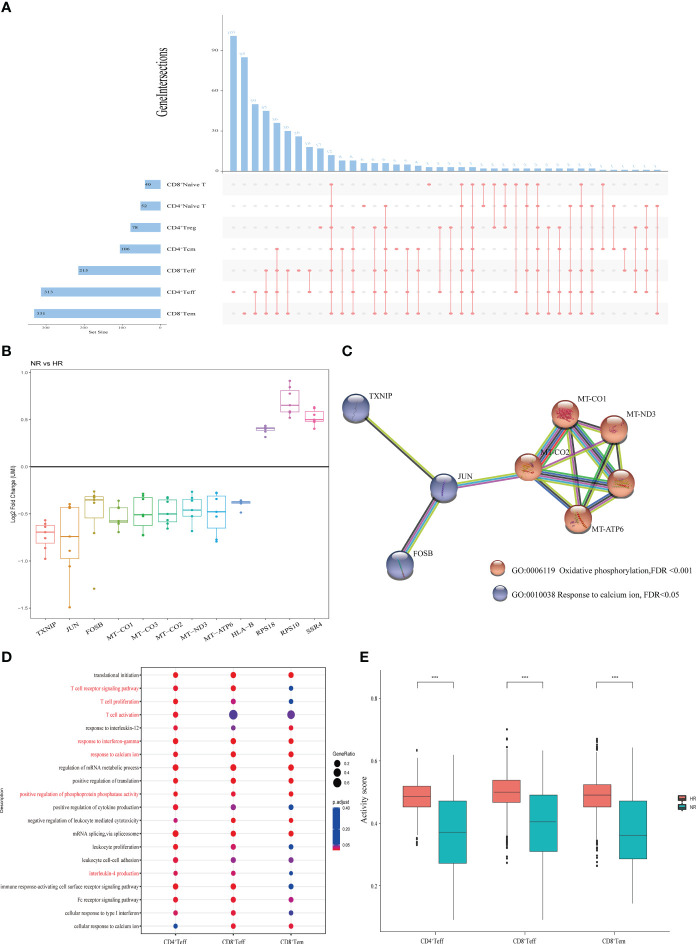
The features of T cell subsets from the non-responder (NR) and high-responder (HR) groups. **(A)** An upset diagram depicting the overlapping and non-overlapping differentially expressed genes (DEGs) in seven T cell subtypes. The horizontal bar chart on the left represents the number of elements in each set, the colored points in the middle and the lines between the points represent the intersection of different T cell subsets, and the vertical bar chart at the top represents the number of corresponding intersection elements. **(B)** A boxplot showing the log2 fold changes of the unique molecular identifier (UMI) counts of 12 DEGs common to the seven T cell subtypes. **(C)** Protein–protein interaction (PPI) network showing the GO terms associated with the 12 common DEGs. **(D)** Enrichment analysis of the downregulated genes in three T cell subtypes with marked differential characteristics; the Gene Ontology (GO) terms of interest are shown in red. **(E)** Cell activity scores of three T cell subtypes. A *P*-value <0.05 was considered significant. ****P<0.0001 (Student’s *t*-test).

As previously shown in **Figure 1**, seven T cell subsets were identified in this study. To investigate the features of adaptive immunity in non-responders, we compared the gene expression profile of each T cell subset between the two groups (**Supplementary Figures 4A–G**). Intersection analysis of the DEGs indicated that the three cell clusters with the highest numbers of DEGs and cell type-specific DEGs were CD8^+^ effector memory T (Tem) cells, CD4^+^ effector T (Teff) cells, and CD8^+^ Teff cells (**Figure 4A**). Meanwhile, 12 DEGs were common to all the T cell subsets, including nine that were downregulated and three that were upregulated (**Figures 4A, B**). A PPI network of the 12 DEGs demonstrated that *TXNIP*, *JUN*, and *FOSB* are involved in calcium ion reactions and are associated with oxidative phosphorylation (**Figure 4C**).

Next, we performed a GO term enrichment analysis on the three above-mentioned T cell subsets (**Figure 4D**). The results showed that all the downregulated genes in these T cell subsets from the NR group were involved in biological processes associated with immune cell activity, including cell activation, calcium ion reaction, interferon-γ, and oxidative phosphorylation. Two genes downregulated in CD4^+^ Teff and CD8^+^ Teff cells—*CEBPB* and *NDFIP1*—were involved in the production of interleukin-4 (IL-4). Other downregulated genes identified (**Supplementary Table 7**) were involved in pathways such as the T cell receptor signaling pathway, the antigen receptor-mediated signaling pathway, and T cell proliferation. Finally, all the cell activity scores of the three T cell subsets were significantly lower in the NR group than in the HR group (**Figure 4E**).

### 3.5 Transcriptional characteristics of natural killer and NKT cells of non-responders

**Figure 5:**
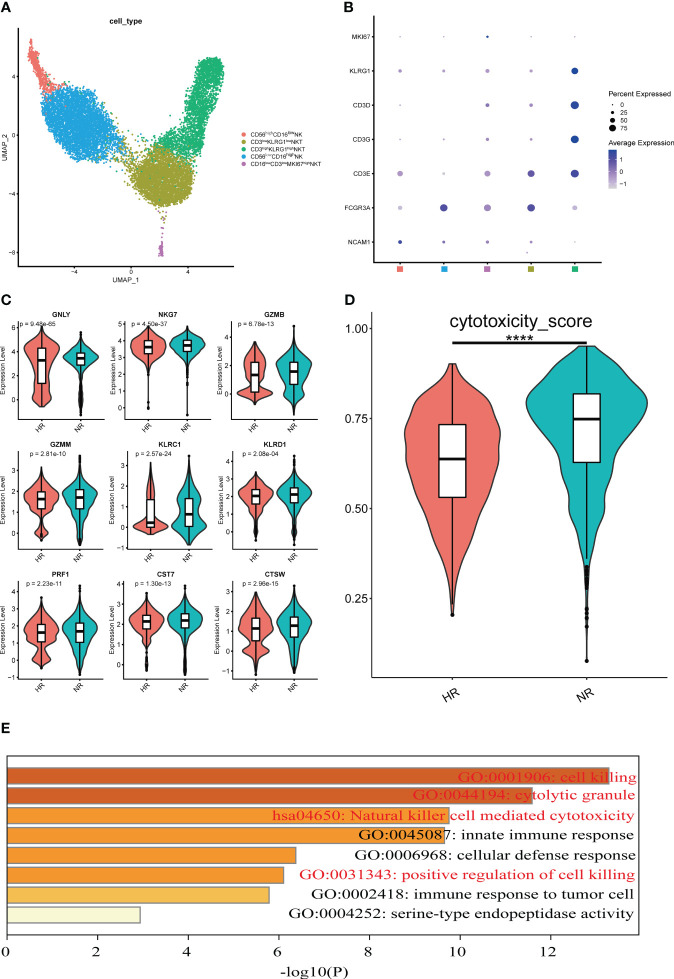
Features of natural killer (NK) and natural killer T (NKT) cell subsets from the non-responder (NR) and high-responder (HR) groups. **(A)** A UMAP displaying five cell subsets identified from 5,615 high-quality NK cells and 6,989 high-quality NKT cells in the NR and HR groups; each dot represents one cell and dots are colored according to the cell subset. **(B)** A dot plot showing the expression level of the selected classical marker genes in five cell subsets; the color of the dots represents the expression level, the size of each dot represents the proportion of cells expressing the corresponding gene, and the squares along the *x*-axis represent the cell subsets corresponding to the subsets in A. **(C)** A violin plot showing the expression levels of nine cytotoxicity-related marker genes with statistical significance in CD3^high^KLRG1^high^ NKT cells; the Wilcox rank-sum test was used for analysis. The horizontal line dividing the box represents the median value and the two ends of the box represent the upper quartile (Q3) and lower quartile (Q1); the difference between Q1 and Q3 represents the interquartile range (IQR). **(D)** The cytotoxicity scores of CD3^high^KLRG1^high^ NKT cells are shown; a *P*-value <0.05 was considered significant. ****P<0.0001 (Student’s *t*-test). **(E)** Enrichment analysis of upregulated genes in CD3^high^KLRG1^high^ NKT cells from the NR group. The Gene Ontology (GO) and Kyoto Encyclopedia of Genes and Genomes (KEGG) terms of interest are shown in red.

According to their transcriptional characteristics, two subsets of NK cells were characterized as CD56^low^CD16^high^ and CD56^high^CD16^low^. Three subsets of NKT cells identified were categorized as CD3^low^CD16^high^, CD3^high^CD16^low^KLRG1^high^, and CD3^low^CD16^low^MKI67^high^ (**Figures 5A, B**). No difference in the proportion of each subset was observed between the NR and HR groups (**Supplementary Figure 5A, B**).

To understand the role of NK and NKT cells in the immune response to the hepatitis B vaccine, the transcriptional characteristics of each cell subset were compared between the two groups (**Supplementary Figure 5C**). CD56^low^CD16^high^ NK cells and CD3^high^CD16^low^KLRG1^high^ NKT cells were the subtypes with the greatest number of cell-type specific DEGs (**Supplementary Figure 5D**). Next, we undertook a comprehensive analysis of the DEGs from the two subtypes. Notably, we found that the relative expression levels of cytotoxicity-associated markers (such as *GNLY*, *NKG7*, *GZMB*, *GZMM*, *KLRC1*, *KLRD1*, *PRF1*, *CST7*, and *CTSW*) in the CD3^high^CD16^low^KLRG1^high^ NKT cell subset were higher in the NR group than in the HR group (**Figure 5C**). Additionally, the cytotoxicity scores of the cluster from the NR group were significantly higher than those in the HR group (**Figure 5D**). Furthermore, we observed that the upregulated DEGs in the NR group were involved in biological processes such as cell killing and NK cell-mediated cytotoxicity (**Figure 5E**).

### 3.6 RT-qPCR verification of the results

**Figure 6:**
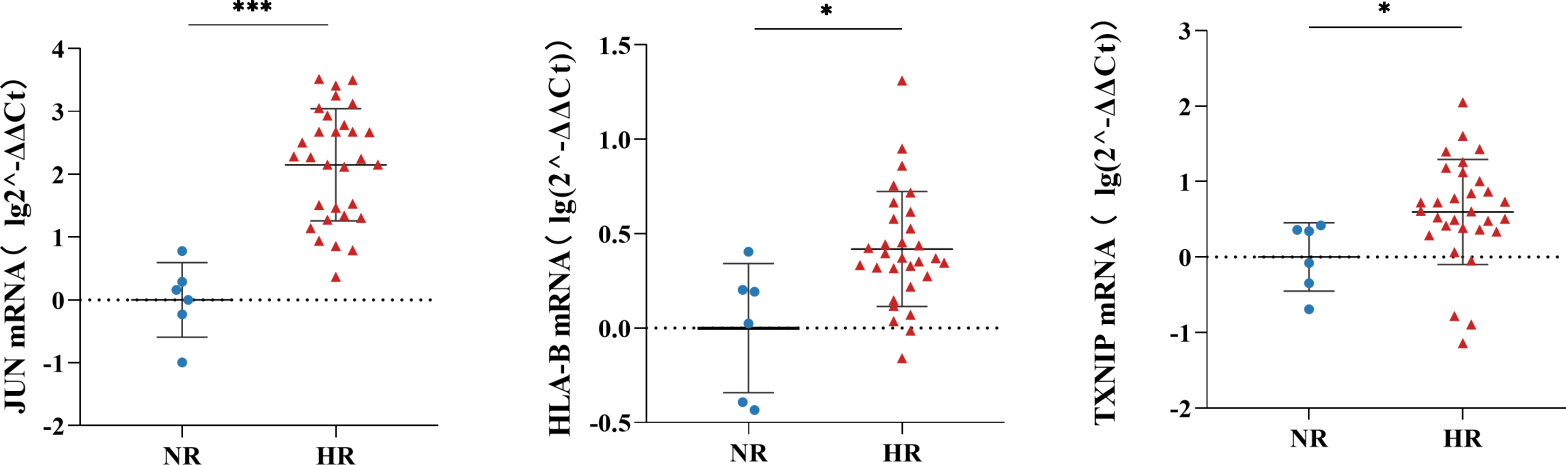
The relative expression levels of JUN, HLA-B, and TXNIP in peripheral blood mononuclear cells (PBMCs) of the non-responder (NR) (*n*=6) and high-responder (HR) (*n*=30) groups; the Mann-Whitney U Test was used to compare differences between independent groups and a *P*-value <0.05 was considered significant. *P<0.05, ***P<0.001.

Given the difference in sensitivity between RT-qPCR and single-cell RNA-seq, we selected three genes—*JUN*, *HLA-B*, and *TXNIP—*that were noticeably downregulated in most cell subtypes in our single-cell RNA-seq analysis for verification by RT-qPCR (**Supplementary Figure 6**). Consistent with the sequencing results, the RT-qPCR analysis showed that these three genes were significantly downregulated in the NR group compared with the HR group (**Figure 6**).

## 4 Discussion

The mechanism underlying the absence of a response to the hepatitis B vaccine is still not fully understood. In this study, through a comparative analysis of single-cell transcriptional characteristics of non-responders and high responders to the vaccine, we have provided potential targets that may contribute to improving the intensity of the immune response to hepatitis B vaccination. Seventeen immune cell types were identified from the peripheral blood of volunteers. Although no differences in cell type composition were found between the HR and NR groups in our study, several studies have reported that the proportion of CD4^+^ Tregs is higher in non-responders than in high responders (16, 17), which may be associated with differences in the protocols used between these studies and ours.

In this study, we demonstrated the heterogeneity of expression of HLA molecules in different APCs at the single-cell level. In addition, except for pDCs, the expression enrichment score of HLA-I molecules in APC sub-clusters was significantly lower in non-responders than in high responders, while the expression levels of HLA-II molecules in the various APC sub-clusters were significantly lower in the NR group than in the HR group. This implied that antigen presentation activity was weaker in the former group than in the latter one. Additionally, the downregulated DEGs in the various APCs derived from the NR group were significantly enriched in the antigen presentation and processing signaling pathways, which again suggested that the antigen processing and presentation functions of APCs are impaired in non-responders. Our results also supported the hypothesis that the peptides of the hepatitis B surface antigen may not be degraded into smaller antigenic peptides because of deficient antigen presentation and processing or, alternatively, that a lack of HLA-I and HLA-II molecules connected to these peptides may lead to functional deficits in APCs (18, 19). Furthermore, our data suggested that HLA-DRB5 and HLA-B may be the key molecules responsible for hepatitis B virus (HBV) antigen presentation, and the low expression of HLA-DRB5 and HLA-B may be one of the main factors influencing the lack of response to the vaccine. Similar to this result, it was reported that HLA-DRB5 is significantly downregulated in the liver tissue of patients with hepatitis B who showed a poor response to antiviral therapy (20). A different study demonstrated that the concentrations of HLA-DR and HLA-ABC antigens, markers for activated mature monocyte-derived dendritic cells (moDCs), were significantly lower in non-responders than in high responders (21).

The number of B cells producing HBsAb was reported to be significantly reduced in non-responders; however, the molecular mechanism underlying this effect remains unclear (22). In our study, the scores for MAPK pathway activity in naive B cells from non-responders were especially low. The MAPK pathway is the hub of the eukaryotic signal transmission network and may play an important role in defending against virus infection (12, 23). Another study showed that the inhibition of the NF-κB and p38 MAPK pathways suppressed the HBsAg-induced secretion of cytokines in moDCs (24). We further found that molecules such as B cell linker protein (BLNK), B lymphoid tyrosine kinase (BLK), interleukin-4 receptor (IL-4R), SLP adaptor and CSK interacting membrane protein (SCIMP), NFKBIA, and JUN were underexpressed in naive B cells from non-responders. BLNK acts as a central linker protein downstream of the B-cell receptor (BCR) and connects spleen-associated tyrosine kinase (SYK) to several signaling pathways (25). Additionally, BLNK plays a crucial role in ERK/EPHB2, p38MAPK, JNK, NF-κB, NFAT, and AP-1 activation, the regulation of B cell function and development (26), and BCR-mediated activation of PLCG1, PLCG2, and Ca2^+^ mobilization (27). BLK, a member of the Src tyrosine kinase family, can exert both positive and negative regulatory effects on B cells (28, 29). IL-4R regulates the expression of numerous genes and promotes cell proliferation and differentiation through the common cytokine receptor γ chain-mediated activation of STAT proteins (30). The low expression of IL-4R in naive B cells in the NR group observed in the present study is in agreement with the results of a study demonstrating that IL-4 secretion is reduced in non-responders to the hepatitis B vaccine (31). SCIMP is expressed in B cells and other professional APCs and undergoes tyrosine phosphorylation under the stimulation of the major histocompatibility complex (MHC) type II signal. Phosphorylated SCIMP then recruits SLP65/SLP76 and GRB2 complexes, thereby activating the PLCγ1/2 and RAS pathways, among others (32–34). NFKBIA can promote its own synthesis by binding to the NF-κB transcription factor, which can block the binding of NF-κB to DNA, thus inhibiting the NF-κB signaling pathway (35). One study reported that the expression of IκBα (encoded by *NFKBIA*) is increased in gastric cancer after EBV infection (36). Combined, these observations suggest that the reduced expression of these molecules in naive B cells in non-responders reduces the activity of the MAPK and NF-κB pathways, consequently weakening the expression of AP-1 family members. These effects may underlie the lack of response to the hepatitis B vaccine.

The recognition of foreign antigens by both CD4^+^ and CD8^+^ T cells is MHC-restricted (37, 38). Like that seen with HLA-I and HLA-II molecules, we found that the expression levels of genes associated with Ca^2+^ signaling and oxidative phosphorylation were reduced in seven subsets of T cells, with *JUN* being the representative gene. c-Jun N-terminal phosphorylation was reported to play an important role in the progression of HBV infection (39). Furthermore, two genes downregulated in CD4^+^ Teff cells in the NR group—*CEBPB* and *NDFIP1*—were associated with IL-4 production, which was in line with a previous report that showed the secretion of IL-4 was reduced in non-responders to the hepatitis B vaccine (31). CCAAT enhancer binding protein beta (CEBPB) can influence the activation of IL-4/IL-13 and NF-κB signaling through transcriptional repression during ER stress (40, 41). Nedd4 family interacting protein 1 (NDFIP1) plays a crucial role in CD4^+^ T cell proliferation and can maintain immune homeostasis by inhibiting the secretion of IL-4 (42). Our results further demonstrated that the expression scores of cell activity-related genes in CD4^+^ Teff cells were significantly reduced in non-responders. Additionally, several studies have suggested that changes in cell activity may affect the immune response to HBV (43, 44).

In this study, we found that CD3^high^CD16^low^KLRG1^high^ NKT cells from the NR group had higher cytotoxicity-related gene set expression scores than those of the HR group, and the upregulated genes were enriched in cytotoxicity-related pathways. The above characteristics are similar to those reported for CD56^dim^ NK cells and KLRG1^+^ NKT cells from patients with chronic hepatitis B, which had specific memory for and stronger cytotoxic effects against HBsAg-pulsed moDCs, as well as proliferative responses to HBV antigen relative to unvaccinated people (45). In addition, it has been shown that the expression scores for these representative cytotoxicity-related gene sets in a subset of NKT cells were significantly higher in patients infected with SARS-CoV-2 than in healthy people (11). There is evidence to support that, on the one hand, vaccines can induce specific immune memory in NK cells, while on the other, NK cells can also indirectly regulate vaccine-induced humoral and adaptive immunity (46, 47). These observations largely support our findings that the increased cytotoxicity of CD3^high^ CD56^low^ KLRG1^high^ NKT cells is a key factor associated with non-response to the hepatitis B vaccine.

In addition to JUN and HLA-B, we found that *TXNIP*, one of the identified DEGs between the NR and HR groups, was downregulated in most cells. One study showed that reducing the expression of *TXNIP* can inhibit hepatitis C virus replication; however, the mechanism responsible for this phenomenon is not clear (48). Another study showed that TXNIP can maintain cells in a higher state of oxidative stress and promote immunoglobulin production (49).

In summary, some key cells and molecules that may regulate the intensity of the immune response to the hepatitis B vaccine were revealed *via* a comparative single-cell analysis of the transcriptional characteristics of PBMCs from non-responders and high responders. A key reason for the small sample in this study is that it is extremely difficult to identify hepatitis B vaccine non-responders. Accordingly, the mechanism relating to the production of HBsAb by these key cells, the roles played by the genes identified in this study in the absence of response to the hepatitis B vaccine, and the ability of CD3^high^CD56^high^KLRG1^high^ NKT cells to fight HBV infection should be further explored through cell- and animal-based experiments.

## Data availability statement

The original contributions presented in the study are publicly available. This data can be found here: https://www.ncbi.nlm.nih.gov/geo/query/acc.cgi?acc=GSE229431.

## Ethics statement

The studies involving human participants were reviewed and approved by Ethics Committee of Lanzhou First People’s Hospital. The patients/participants provided their written informed consent to participate in this study.

## Author contributions

All the authors contributed to sample collection and the isolation of peripheral blood mononuclear cells. MZ analyzed the single-cell data and performed RT-qPCR. MZ, CW, PL, TS, and XDX participated in the design of the study. MZ and XDX wrote the manuscript. All authors contributed to the article and approved the submitted version.
