# Supplementary material for: Single-cell RNA sequencing reveals the transcriptomic characteristics of peripheral blood mononuclear cells in hepatitis B vaccine non-responders
Source: Front Immunol. 2023 Aug 1;14:1091237. doi: 10.3389/fimmu.2023.1091237 (PMC10431960; doi:10.3389/fimmu.2023.1091237)
Supplement: Supplementary file 3 [file DataSheet_3.zip › Supplementary Methods.DOCX]

**The serum markers of hepatitis B**

The serum markers of hepatitis B include Aspertate Aminotransferase(AST), Alanine aminotransferase(ALT), Hepatitis B surface antigen(HBsAg), Hepatitis B surface antibody(HBsAb), Hepatitis B e antigen(HBeAg), Hepatitis B e antibody(HBeAb), Hepatitis B core antibody and Hepatitis B virus DNA(HBV-DNA).

**Detection instruments and reagents for serum markers**

The serum markers of hepatitis B were detected by Roche electrochemiluminescence instrument(Cobas601)and Roche kit, and hepatitis B virus DNA was detected by real-time fluorescent quantitative PCR instrument and Sanxiang hepatitis B virus DNA quantitative detection kit.

**Details of RT–qPCR**

**Methods for reverse transcription of cDNA**

**Configuration of genome removal system:** the genome removal reaction mixture was prepared according to **Table 1**. In order to ensure accuracy, the genome removal mixture should be prepared according to the amount of reaction number +1, and then packed into each reaction tube. Finally, RNA samples were added to the system (the recommended amount of total RNA added to the system is 0.5ng-5ug)

**Table 1** Components of the genome system removed

| Component | Dosage |
| --- | --- |
| RNA template | 8μL |
| gDNA remover | 1μL |
| 10X gDNA remover Buffer | 1μL |
| RNase-free water | up to 10μL |

**Procedures for removing gDNA:** 42°C for 2min, 60°C for 5min. At the end of the reaction, the RNA template were cooled on ice and 10ul of reverse transcription mixture configured according to **Table 2** was added after a brief centrifugation.

**Table 2** Components of the reverse transcription mixture

| Components | Dosage |
| --- | --- |
| dNTP mix | 1μL |
| Randomer | 1μL |
| 5xGoldenstarTM Buffer | 4μL |
| DTT | 1μL |
| GoldenstarTM | 1μL |
| RNase-free water | 2μL |

**Reverse transcription program：**25°C for 10min, 55℃ for 15min, 85°C for 5min, after the end of reverse transcription, the samples were placed on ice or stored in a -20°C refrigerator for use in the real-time PCR.

**Procedure of the real-time fluorescent quantitative PCR**

**Configuration of the real-time PCR amplification mixture:** Three detection replicates were set for each gene of each sample, and the amplification mixture was configured according to the **Table 3.**

**Table 3 Components of the real-time fluorescence quantitative PCR amplification mixture**

| Components | Dosage |
| --- | --- |
| Master qPCR Mix | 10μL |
| 10uM forward primers | 0.8μL |
| 10uM reverse primer | 0.8μL |
| ROX II | 0.4μL |
| DNA template | 1μL |
| dd H2O | 7μL |
| Total | 20μL |

**Amplification procedures of the real-time fluorescence quantitative PCR:** 95℃ for 1min; 40 cycles (95℃, 10S; 60℃, 30s; fluorescence was collected at 60℃). After the amplification reaction, the meltdown curve of PCR products was established following 95℃ for 15s, 60℃ for 60s and 95℃1s.

**The methods to quantify the expression of target genes were as follows:** (1)Calculating the Ct mean value corresponding to target gene in each sample (2) The Ct mean value of target gene in each sample subtracts the Ct mean value of reference gene in the same sample to obtain the ∆Ct value of target gene in each group.(3)The arithmetic mean of ∆Ct of target gene in all samples of the control group was calculated separately.(4)The ∆Ct mean value of target molecule of each sample in each group subtracts the ∆Ct mean value of the target molecule of all samples in the control group to get the ∆∆Ct value of each sample target gene in each group. (5) The ΔΔCt values of target molecule of each sample in each group obtained in step 4 were calculated by 2^-ΔΔCt to finally obtain the corresponding relative expression quantity of target molecule in each sample in each group.
