# Supplementary material for: Single-cell RNA sequencing reveals the transcriptomic characteristics of peripheral blood mononuclear cells in hepatitis B vaccine non-responders
Source: Front Immunol. 2023 Aug 1;14:1091237. doi: 10.3389/fimmu.2023.1091237 (PMC10431960; doi:10.3389/fimmu.2023.1091237)
Supplement: Supplementary file 3 [file DataSheet_3.zip › Figure 1A-F.DOCX]

**
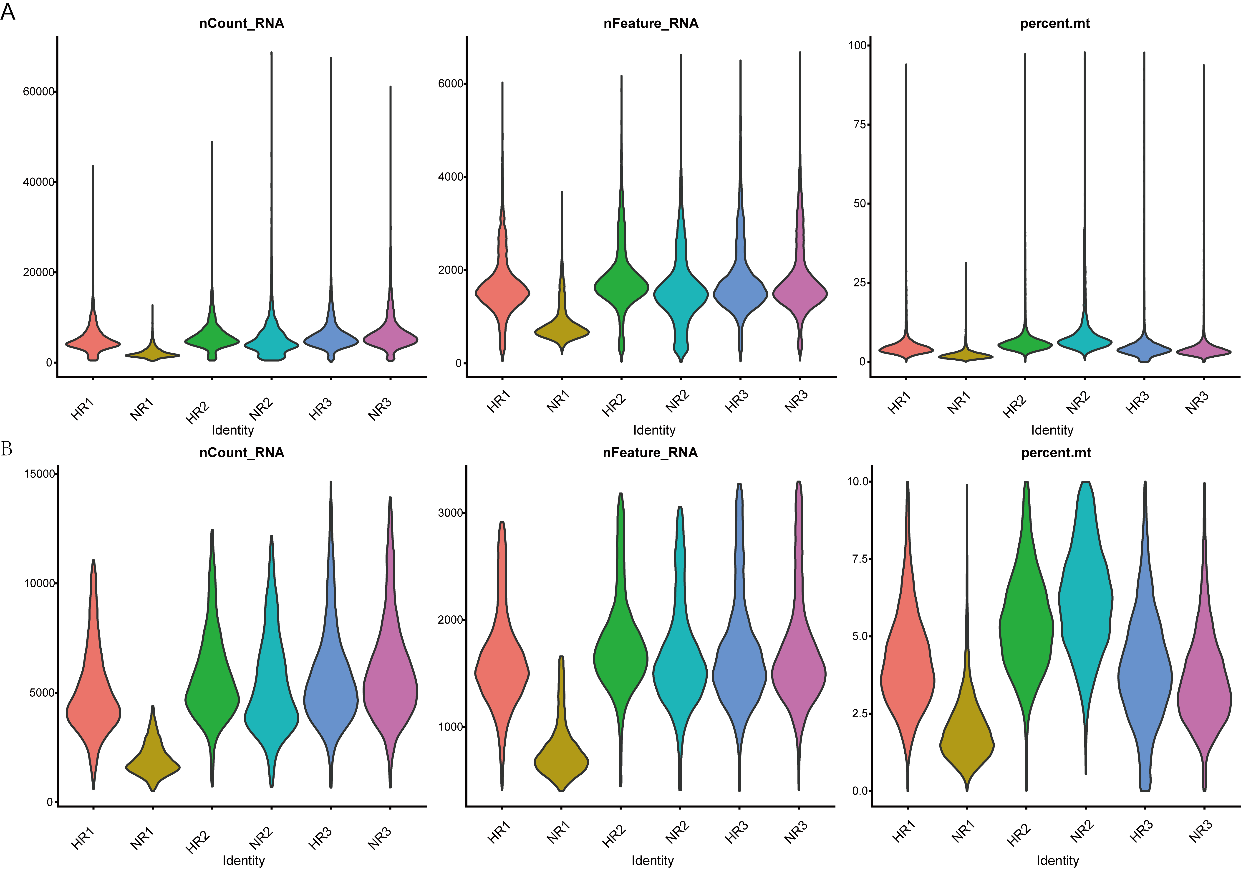
**

**
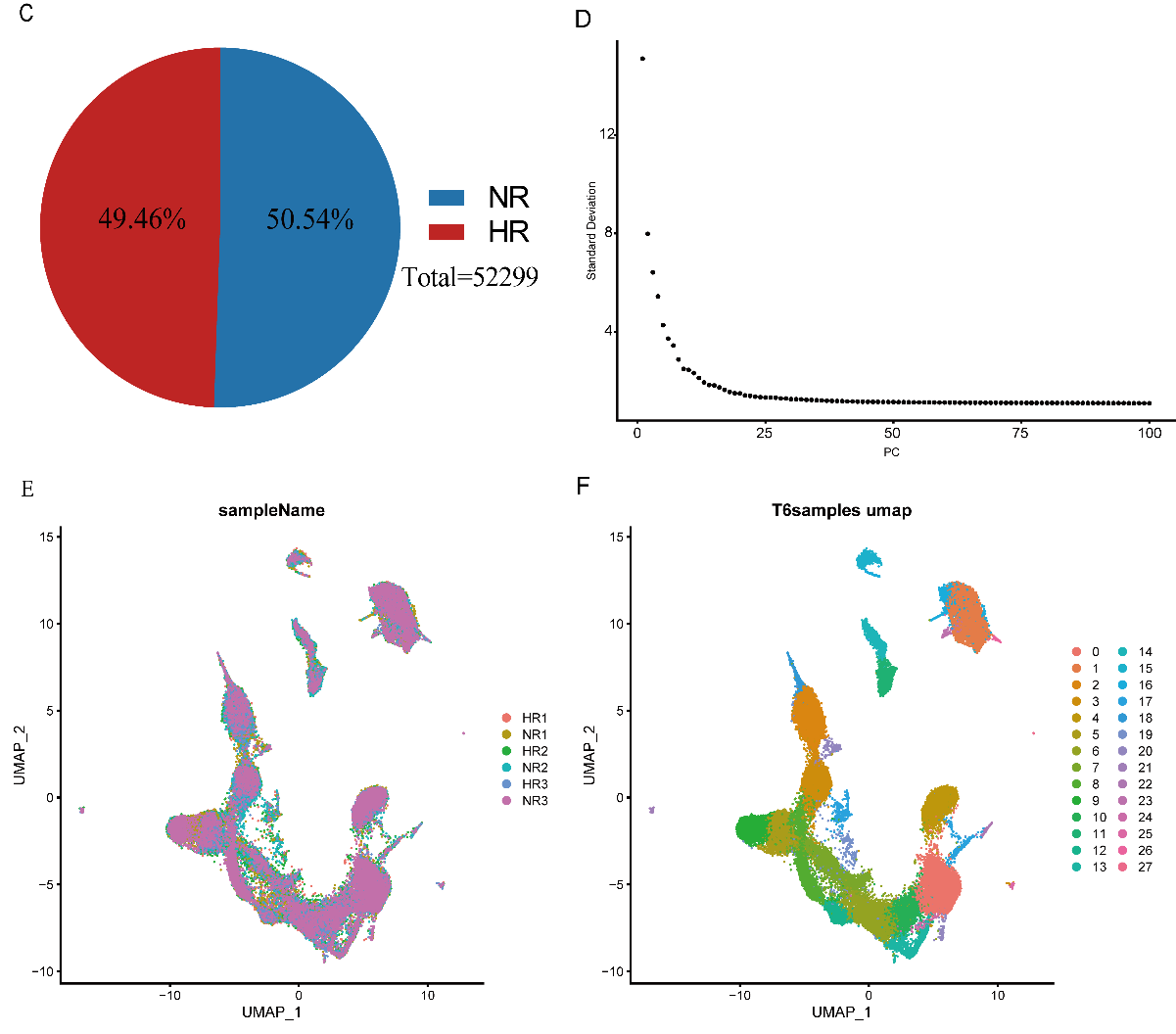
**

**Supplementary Fig 1A-F.** The basic information of single-cell sequencing data. **(A)** A violin plot showing the number of unique molecular identifiers (UMIs) (left), number of genes (middle) and percentage of mitochondrial genes (right) before quality control of sequencing data. **(B)** The violin plot showed the number of UMI (left figure), number of genes (middle) and percentage of mitochondrial genes (right) after quality control of sequencing data. **(C)** A pie charts showing the number of cells captured by single-cell sequencing in NR group and HR group after quality control of sequencing data. **(D)** A elbow plot showing the principal components after quality control of sequencing data, top50 principal components were selected to clustering. (E**, F)** The UMAP plot showing the two mensional spatial distribution of 28 cell types and subtypes after clustering.
