# Supplementary material for: Single-cell RNA sequencing reveals the transcriptomic characteristics of peripheral blood mononuclear cells in hepatitis B vaccine non-responders
Source: Front Immunol. 2023 Aug 1;14:1091237. doi: 10.3389/fimmu.2023.1091237 (PMC10431960; doi:10.3389/fimmu.2023.1091237)
Supplement: Supplementary file 3 [file DataSheet_3.zip › Figure 1G.DOCX]

**
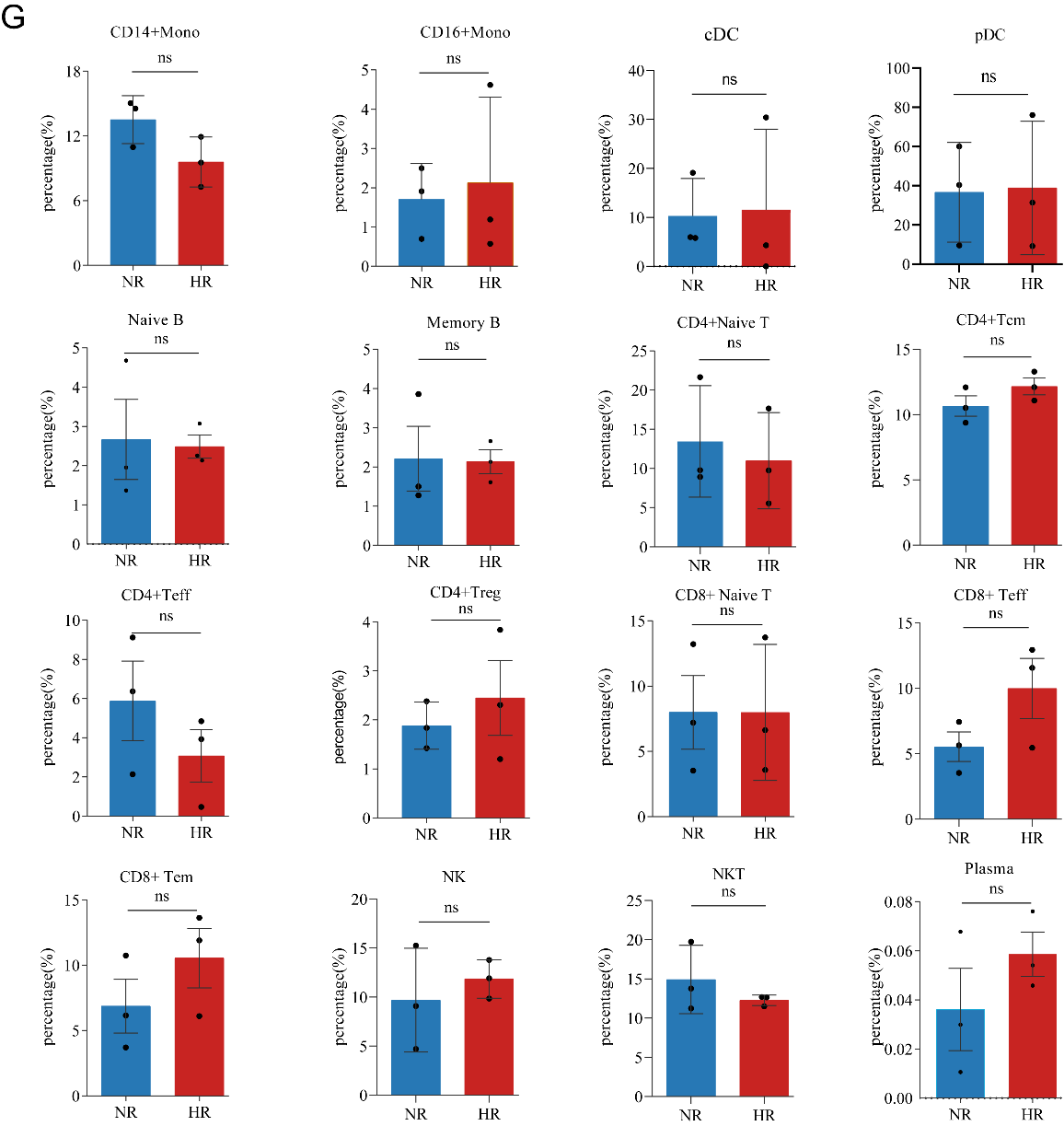
**

**Supplementary Fig 1G.** The percentage of cell number of each cell subgroup in NR group and HR group, ns indicated there was no difference between NR and HR group, the Mann-Whitney U Test in the non-parametric test of independent samples was used for difference analysis.
