# Supplementary material for: Single-cell RNA sequencing reveals the transcriptomic characteristics of peripheral blood mononuclear cells in hepatitis B vaccine non-responders
Source: Front Immunol. 2023 Aug 1;14:1091237. doi: 10.3389/fimmu.2023.1091237 (PMC10431960; doi:10.3389/fimmu.2023.1091237)
Supplement: Supplementary file 3 [file DataSheet_3.zip › Figure 2.DOCX]

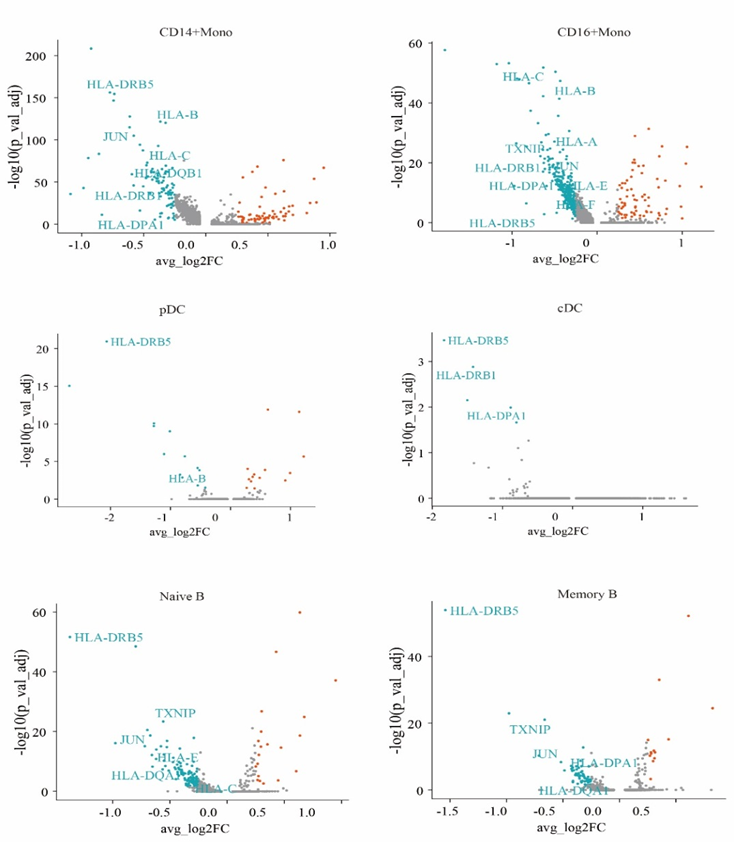


**Supplementary Fig 2. The DEGs of each APC subset from NR group.** Volcano Plot showing the differential genes analyzed by Wilcox rank-sum test (NR VS HR), adjusted p-values <0.05 and |avg_log2FC| > 0.25 represent the difference was statistically significant, the blue dots represent the down-regulated genes in each APC subset from NR group, the red dots represent up-regulated genes in each APC subset from NR group, and the interesting gene is labeled with gene symbol.
