# Supplementary material for: Single-cell RNA sequencing reveals the transcriptomic characteristics of peripheral blood mononuclear cells in hepatitis B vaccine non-responders
Source: Front Immunol. 2023 Aug 1;14:1091237. doi: 10.3389/fimmu.2023.1091237 (PMC10431960; doi:10.3389/fimmu.2023.1091237)
Supplement: Supplementary file 3 [file DataSheet_3.zip › FIgure 3A-D.DOCX]

**
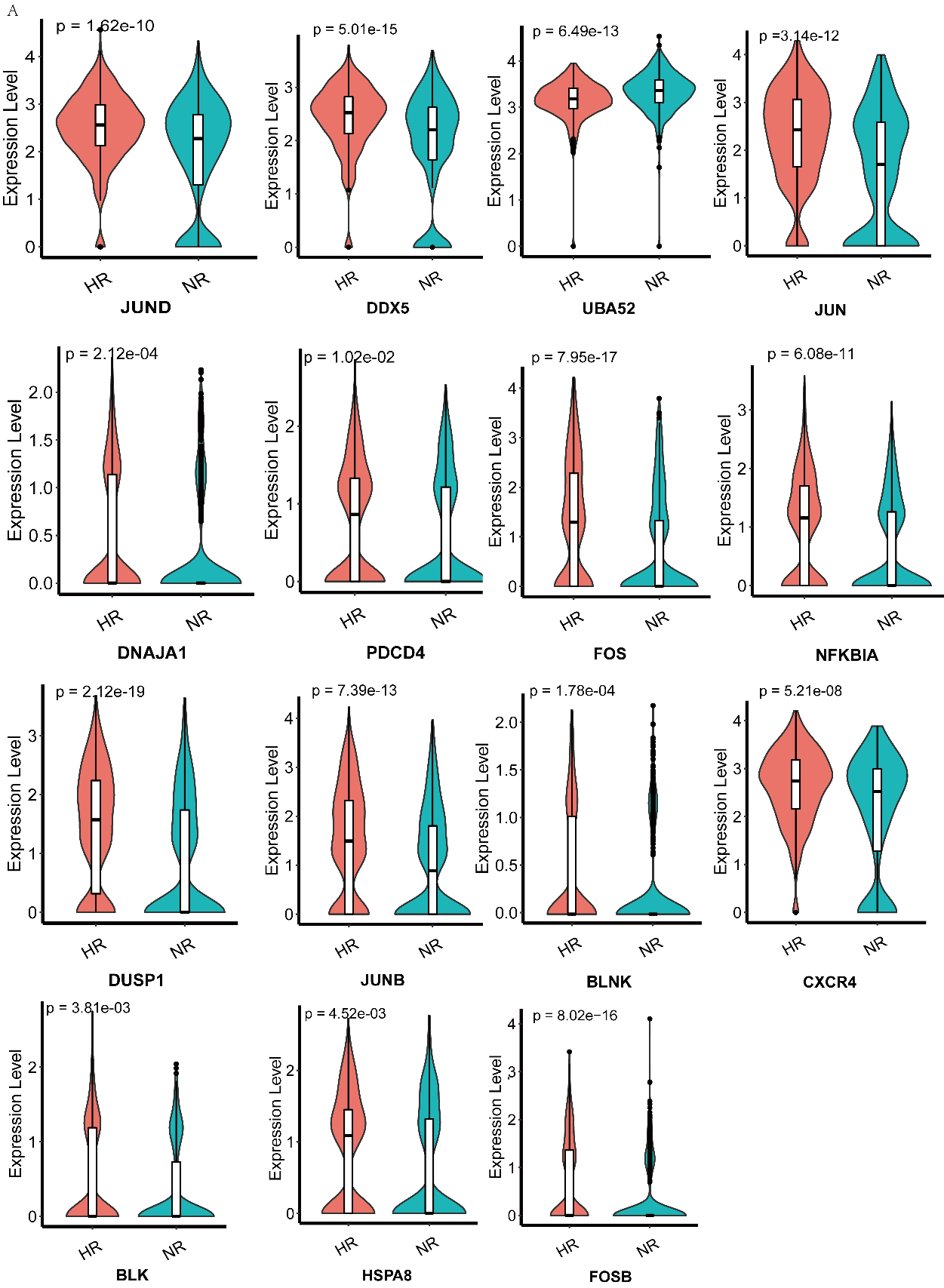
**

**Supplementary Fig 3A.** The expression levels of MAPK-related differential genes in naive B cells of the NR and HR groups was shown by violin plot. The horizontal line dividing the box into two parts represent the median value, the two ends of the box represent the upper quartile (Q3) and the lower quartile (Q1), the difference between quartile Q1 and Q3 is called interquartile range (IQR). All difference with P＜0.05 are indicated, Wilcox test was used for analysis.

**
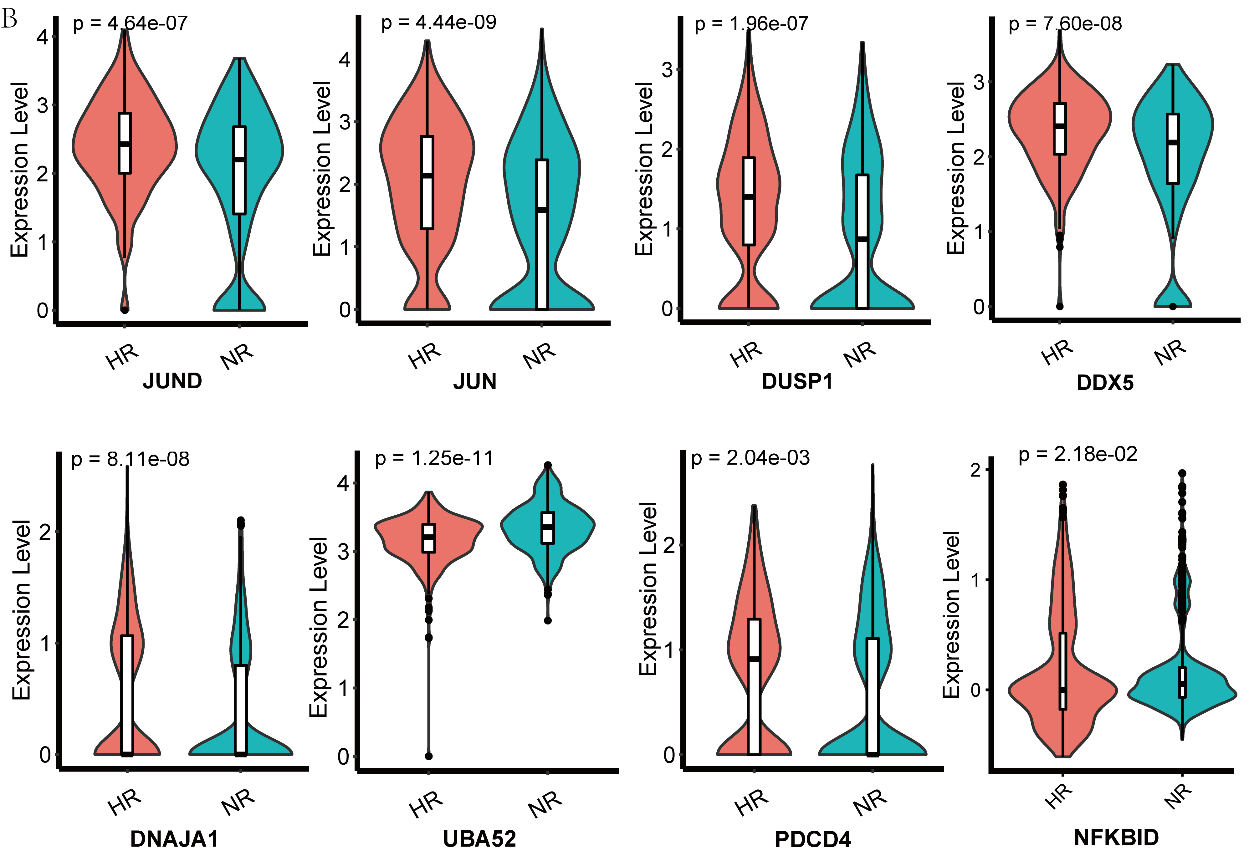
**

**Supplementary Fig 3B.** The expression levels of MAPK-related differential genes in memory B cells of the NR and HR groups was shown by violin plot. The horizontal line dividing the box into two parts represent the median value, the two ends of the box represent the upper quartile (Q3) and the lower quartile (Q1), the difference between quartile Q1 and Q3 is called interquartile range (IQR). All difference with P＜0.05 are significant, Wilcox test was used for analysis.


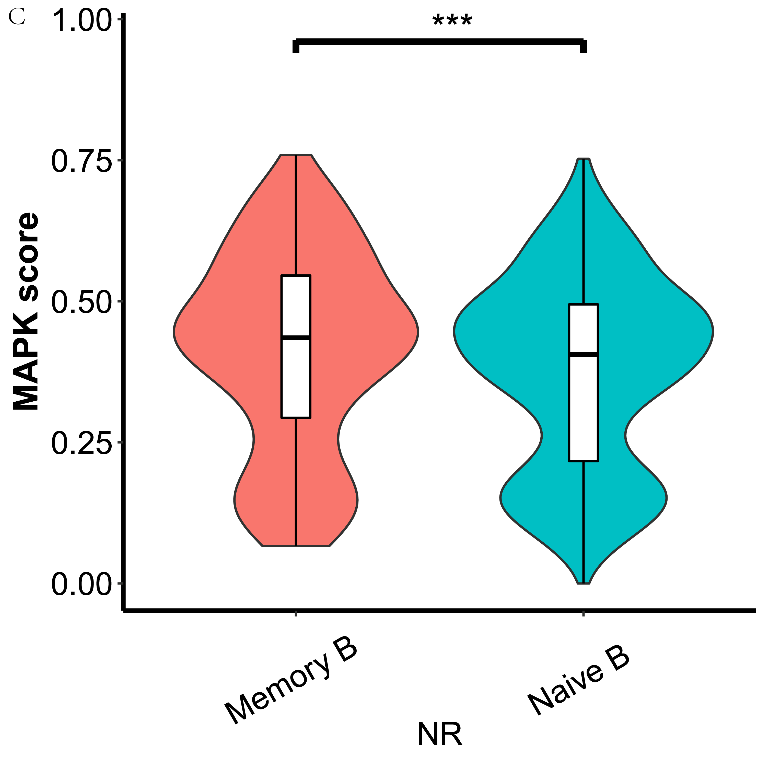


**Supplementary Fig 3C.** A violin plot showing the expression enrichment score of MAPK gene set of memory B and naive B cells in NR group, two independent sample T test was used ,the difference with P＜0.05 are significant. ***p<0.001.


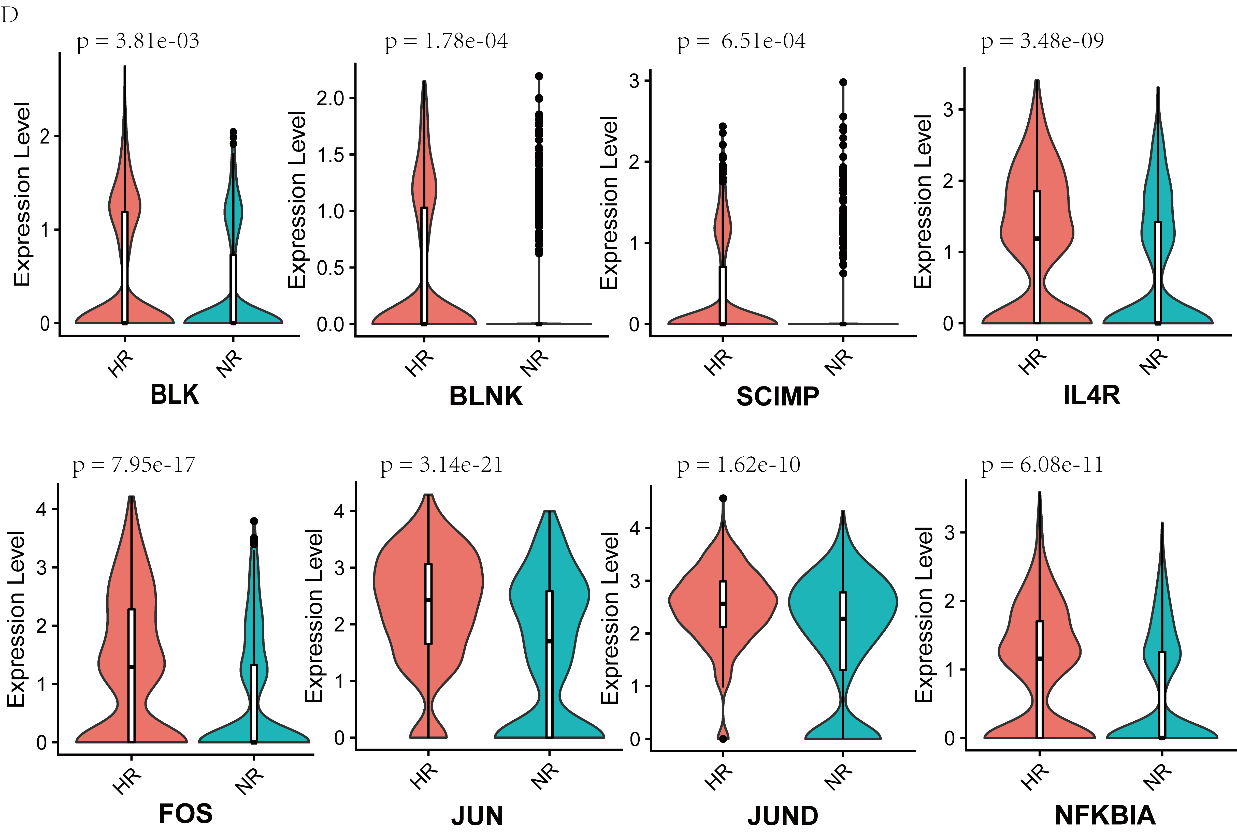


**Supplementary Fig 3D.** The expression level of gene located the downstream of the B cell and IL4R receptor signaling pathways and participated in Ca^2+^, MAPK and NFκB signaling pathways in naive B subset from NR and HR group was show by violin plot.
