# Supplementary material for: Single-cell RNA sequencing reveals the transcriptomic characteristics of peripheral blood mononuclear cells in hepatitis B vaccine non-responders
Source: Front Immunol. 2023 Aug 1;14:1091237. doi: 10.3389/fimmu.2023.1091237 (PMC10431960; doi:10.3389/fimmu.2023.1091237)
Supplement: Supplementary file 3 [file DataSheet_3.zip › Figure 4A-G.DOCX]

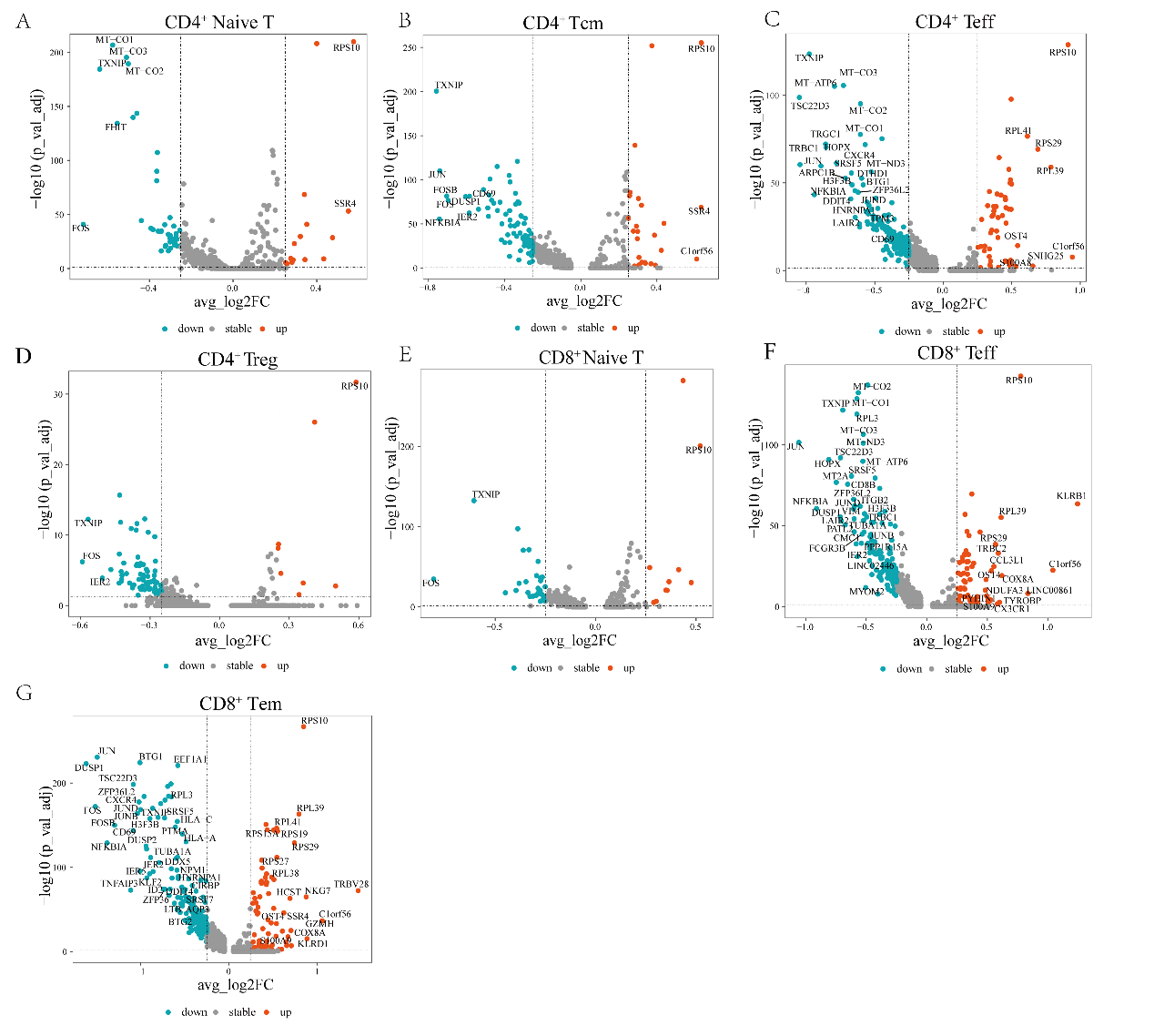


**Supplementary Fig 4A-G.** **The DEGs of each T cell subset from NR group compared to HR group**. Volcano Plots showing the differential genes analyzed by Wilcox rank-sum test (NR group vs. the HR group), adjusted *p*-values <0.05 and |avg_log2FC| > 0.25 represent the difference was statistically significant, the blue dots represent the down-regulated genes in each T cell subset from NR group, the red dots represent up-regulated genes in each T cell subset from NR group, and the gene with adjusted *p*-values < 0.05 and | avg_log2FC | > 0.5 is labeled with gene symbol. The plot A, B, C, D, E, F and G exhibited orderly the DEGs of CD4^+^Naive T, CD4^+^Tcm, CD4^+^Teff, CD4^+^Treg, CD8^+^Naive T, CD8^+^Teff, and CD8^+^TEM cell cluster in NR group.
