# Supplementary material for: Single-cell RNA sequencing reveals the transcriptomic characteristics of peripheral blood mononuclear cells in hepatitis B vaccine non-responders
Source: Front Immunol. 2023 Aug 1;14:1091237. doi: 10.3389/fimmu.2023.1091237 (PMC10431960; doi:10.3389/fimmu.2023.1091237)
Supplement: Supplementary file 3 [file DataSheet_3.zip › Figure 5A-D.DOCX]

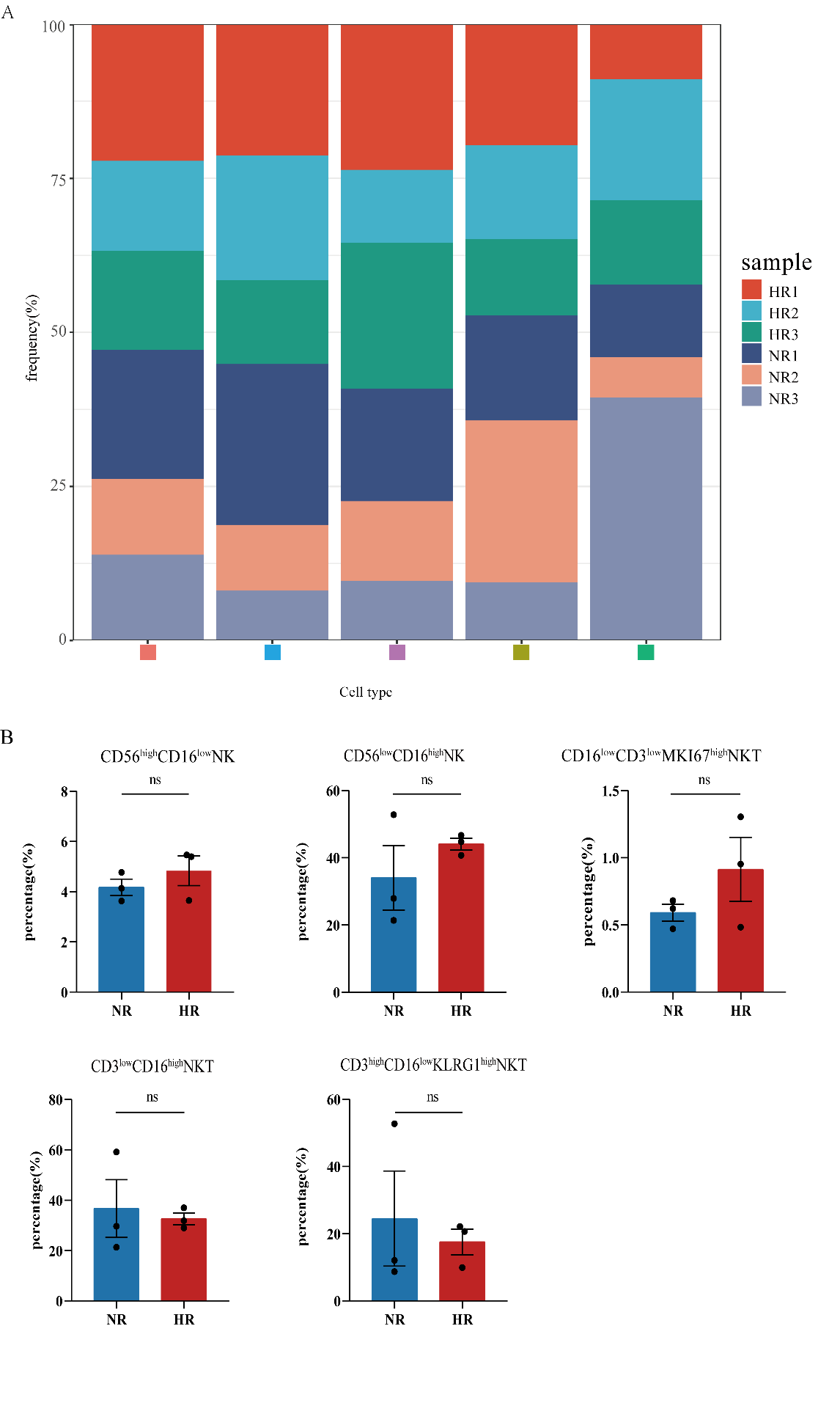


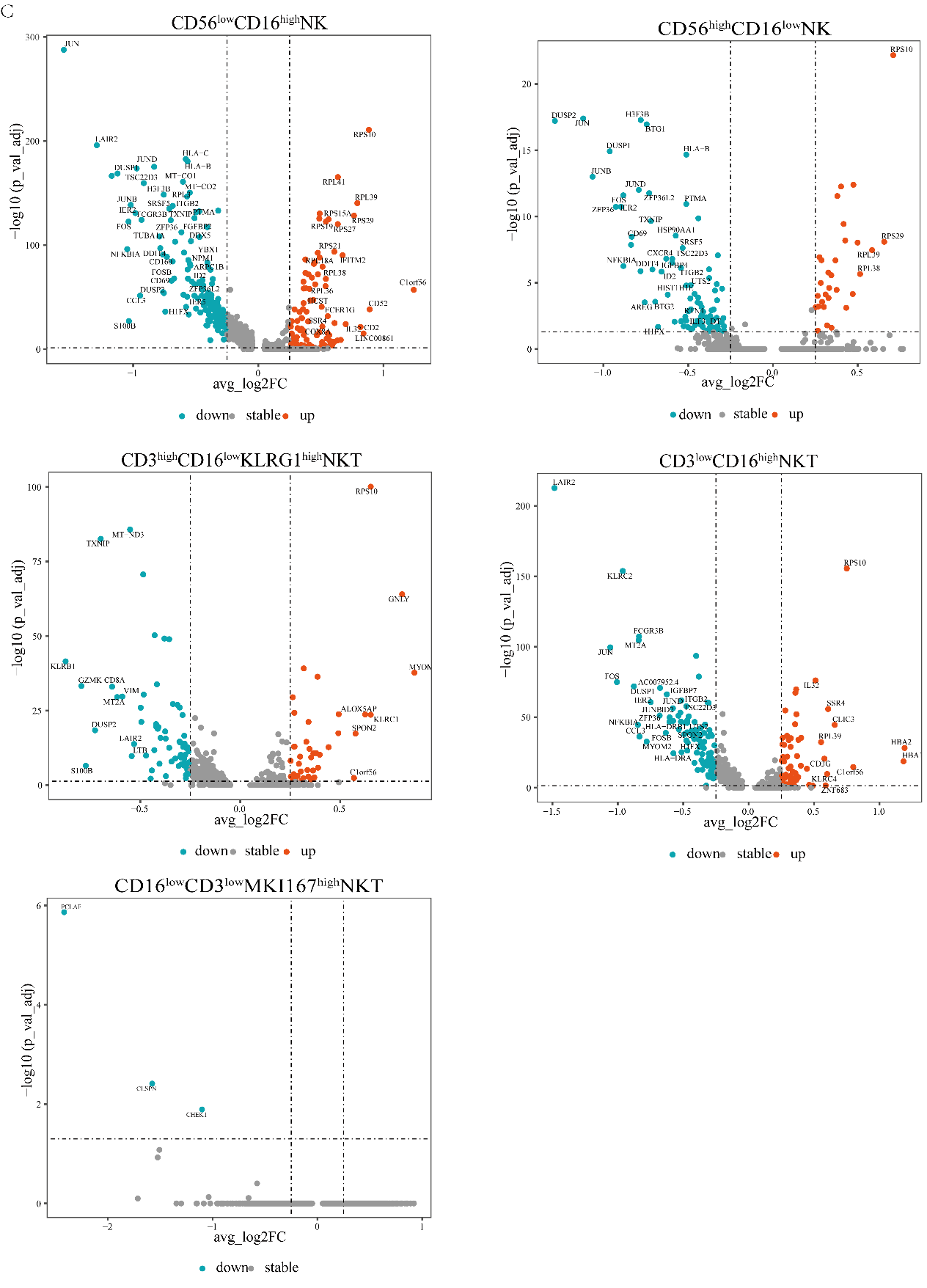


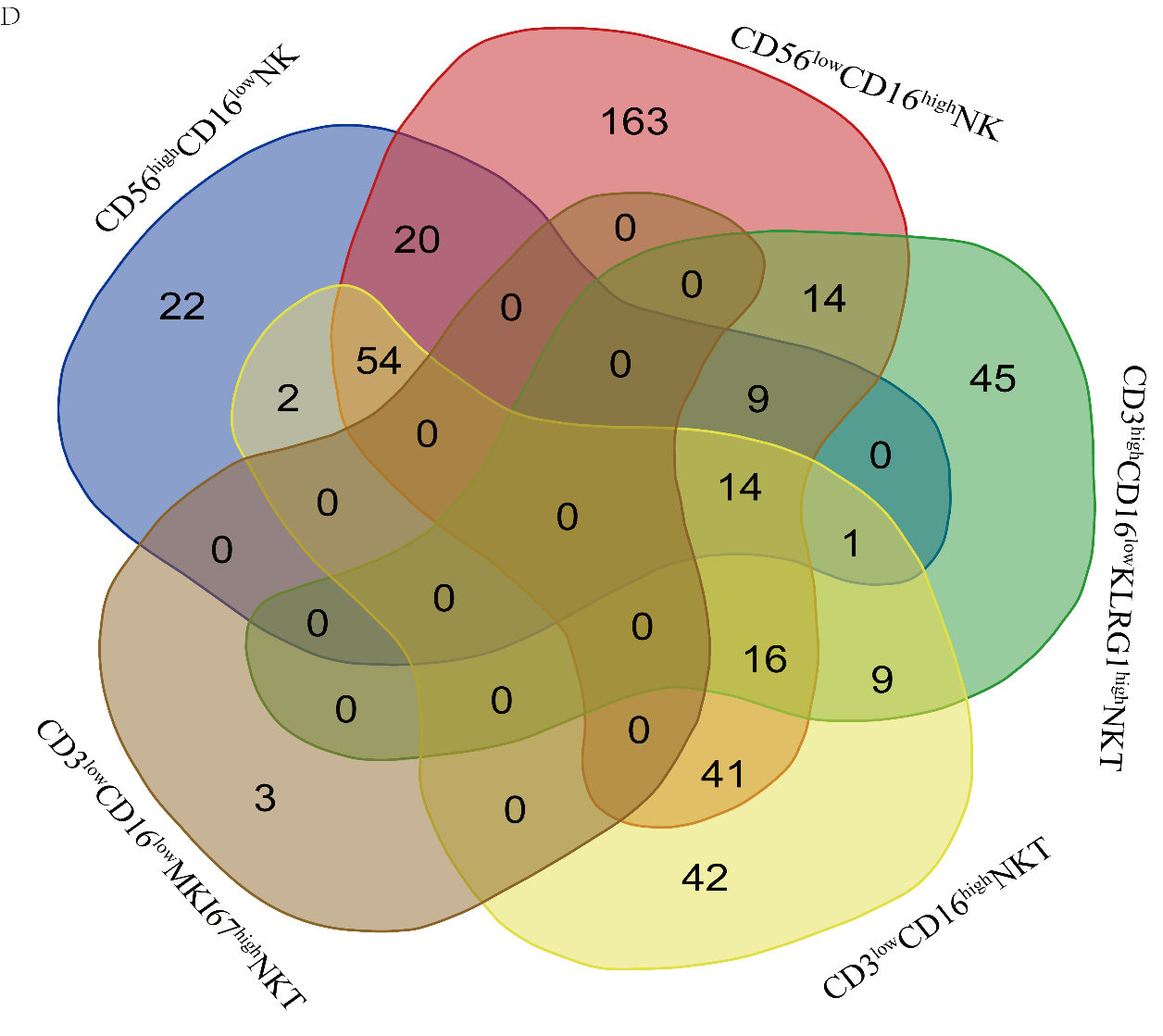


**Supplementary figure 5**. Features of NK and NKT subsets from people with high immune response (HR) and no immune response (NR) to hepatitis B vaccine. **(A)** A stacked bar diagram shows the proportion of each cell type from each sample, the squares along the X-axis representing cell subsets corresponding to cell subsets with the same color in Figure 5a. **(B)** A barplot plots showing the percentages of each cell type in the NR or HR groups, Student t test was used for analysis, ns presents the difference was not statistically significant. **(C)** A volcano plot showing the differential genes analyzed by Wilcox rank-sum test (NR group vs. the HR group), the name of cell subsets are labeled at the top, adjusted *p*-values <0.05 and |avg_log2FC| > 0.25 represent the difference was statistically significant, the blue dots represent the down-regulated genes in NK or NKT subsets from NR group, the red dots represent up-regulated genes in NK or NKT subsets from NR group, and the gene with adjusted *p*-values < 0.05 and | avg_log2FC | > 0.5 is marked with gene symbol. **(D) A** venn diagram showing the number of overlapping differential genes and cell type-specific differential genes in NK and NKT cell subsets from NR group.
