# Supplementary material for: Single-cell RNA sequencing reveals the transcriptomic characteristics of peripheral blood mononuclear cells in hepatitis B vaccine non-responders
Source: Front Immunol. 2023 Aug 1;14:1091237. doi: 10.3389/fimmu.2023.1091237 (PMC10431960; doi:10.3389/fimmu.2023.1091237)
Supplement: Supplementary file 3 [file DataSheet_3.zip › Figure 6A-D.DOCX]

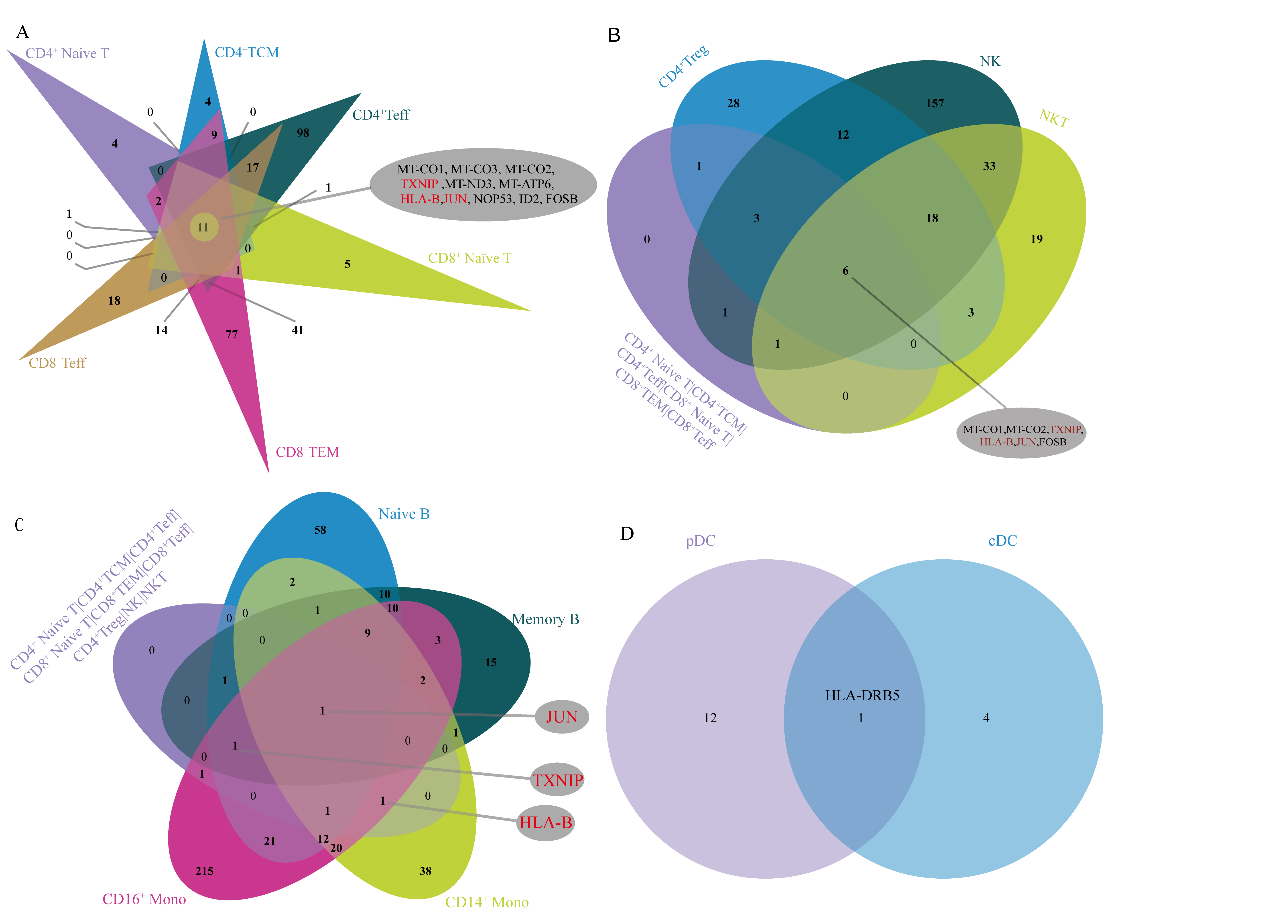


Supplementary Fig 6. Venn diagram shows overlapping low expression genes among cell clusters, three genes in red that were low expressed in cell clusters of more than 12 and interest us were selected. **(A)** A venn plot showing the overlapping low expression genes between CD4^+^Naive T, CD4^+^Tcm, CD4^+^Teff, CD8^+^Naive T, CD8^+^Teff, and CD8^+^TEM. **(B)** A venn plot showing the overlapping low expression genes between all T cell cluster, NK and NKT. **(C)** A venn plot showing the overlapping low expression genes between all T cell cluster, NK, NKT, CD14^+^Mono and CD16^+^Mono. **(D)** A venn plot showing the overlapping low expression genes between pDc and cDC subsets.
