# Supplementary material for: Single-cell RNA sequencing reveals the transcriptomic characteristics of peripheral blood mononuclear cells in hepatitis B vaccine non-responders
Source: Front Immunol. 2023 Aug 1;14:1091237. doi: 10.3389/fimmu.2023.1091237 (PMC10431960; doi:10.3389/fimmu.2023.1091237)
Supplement: Supplementary file 3 [file DataSheet_3.zip › Table 1.DOCX]

**Supplementary Table 1**. Basic characteristic of 6 volunteers

| Parameter | HR1 | HR2 | HR3 | NR1 | NR2 | NR3 |
| --- | --- | --- | --- | --- | --- | --- |
| Race | Chinese Hui population | Chinese Han population | Chinese Han population | Chinese Hui population | Chinese Han population | Chinese Han population |
| Age(Y) | 54 | 29 | 25 | 54 | 32 | 26 |
| Gender | Male | Male | Male | Male | Male | Male |
| Body mass index(BMI) | 22.8 | 19.6 | 19.8 | 23.8 | 21.3 | 19.5 |
| underlying disease | No | No | No | No | No | No |
| AST | 30 U/L | 16 U/L | 12 U/L | 7 U/L | 13 U/L | 22 U/L |
| ALT | 24 U/L | 20 U/L | 16 U/L | 15 U/L | 21 U/L | 26 U/L |
| HBsAg | negative | negative | negative | negative | negative | negative |
| HBsAb | negative | negative | negative | negative | negative | negative |
| HBeAg | negative | negative | negative | negative | negative | negative |
| HBeAb | negative | negative | negative | negative | negative | negative |
| HBcAb | negative | negative | negative | negative | negative | negative |
| HCV | negative | negative | negative | negative | negative | negative |
| HEV | negative | negative | negative | negative | negative | negative |
| HIV | negative | negative | negative | negative | negative | negative |
| HBV-DNA | <5.0E+02 | <5.0E+02 | <5.0E+02 | <5.0E+02 | <5.0E+02 | <5.0E+02 |
| HBsAb（mIU/ml） | ≥1000 | ≥1000 | ≥1000 | <10 | <10 | <10 |
