# Supplementary material for: Single-cell RNA sequencing reveals the transcriptomic characteristics of peripheral blood mononuclear cells in hepatitis B vaccine non-responders
Source: Front Immunol. 2023 Aug 1;14:1091237. doi: 10.3389/fimmu.2023.1091237 (PMC10431960; doi:10.3389/fimmu.2023.1091237)
Supplement: Supplementary file 3 [file DataSheet_3.zip › Table 2.DOCX]

**Supplementary Table 2.** The marker genes of 17 cell types

| Cell types | Marker genes |
| --- | --- |
| Naive B cell | CD79A, CD79B, MS4A1, IGHD, CD38 |
| Memory B cell | CD79A, CD79B, MS4A1, IGHD, CD27 |
| CD14^+^ monocyte cell (CD14+ mono) | CD14 |
| CD16^+^ monocyte cell (CD16+ mono) | CD16 |
| CD4^+^Naive T cell | CD4, TCF7, LEF1 |
| CD4^+^ Central memory T cell (CD4+ Tcm) | CD4, IL7R, CD27, LTB |
| CD4^+^ effector T cell (CD4+ Teff) | CD4, GZMH, GNLY |
| CD4^+^ regulation T cell (CD4+Treg) | CD4, IL2RA, FOXP3, CTLA4 |
| CD8^+^ Naive T cell | CD8, TCF7, LEF1 |
| CD8^+^ effector memory T cell (CD8+ Tem) | CD8B, IL7R, GZMK |
| CD8^+^ effector T cell (CD8+ Teff) | CD8B, GZMH, GNLY |
| classical dendritic cells (cDC) | CD1C, CLEC10A |
| plasmacytoid dendritic cell (pDC) | JCHAIN, SERPINF |
| Plasma | IGHM, JCHAIN |
| natural killer cell (NK) | NCAM1, KLRF1, KLRB1 |
| natural killer T cell (NKT) | CD3E, NCAM1, KLRF1 |
| Patelets | PF4, PPBP |
