# Supplementary material for: Single-cell RNA sequencing reveals the transcriptomic characteristics of peripheral blood mononuclear cells in hepatitis B vaccine non-responders
Source: Front Immunol. 2023 Aug 1;14:1091237. doi: 10.3389/fimmu.2023.1091237 (PMC10431960; doi:10.3389/fimmu.2023.1091237)
Supplement: Supplementary file 3 [file DataSheet_3.zip › Table 3.DOCX]

Supplementary Table 3. Parameter information for the differential gene of interest in APC from NR group compared with HR group

| Gene | Cluster | P_val | avg_log2FC | pct.1^[[1]](#footnote-1)^ | pct.2^[[2]](#footnote-2)^ | P_val_adj | Change^[[3]](#footnote-3)^ |
| --- | --- | --- | --- | --- | --- | --- | --- |
| HLA-DRB5 | CD14 | 9.80E-59 | -1.540863 | 0.143 | 0.585 | 1.23E-54 | low |
|  | CD16 | 1.22E-07 | -0.621826 | 0.205 | 0.347 | 0.0015274 | low |
|  | pDC | 8.75E-26 | -2.064319 | 0.316 | 0.918 | 1.1E-21 | low |
|  | cDC | 2.73E-08 | -1.831824 | 0.571 | 1 | 0.0003428 | low |
|  | Naive B | 1.95E-56 | -1.373288 | 0.132 | 0.537 | 2.45E-52 | low |
|  | Memory B | 9.80E-59 | -1.540863 | 0.143 | 0.585 | 1.23E-54 | low |
| HLA-B | CD14 | 5.27E-125 | -0.323891 | 0.992 | 0.998 | 6.61E-121 | low |
|  | CD16 | 3.42E-46 | -0.444835 | 0.998 | 1 | 4.29E-42 | low |
|  | pDC | 1.15E-08 | -0.517463 | 1 | 1 | 0.0001448 | low |
|  | cDC | 9.05E-04 | -0.437467 | 1 | 1 | 1 | no |
|  | Naive B | 2.21E-07 | -0.223398 | 0.975 | 0.994 | 0.0027726 | no |
|  | Memory B | 4.76E-08 | -0.208979 | 0.996 | 1 | 0.0005971 | no |

1. The proportion of this gene expressed in all cells of the corresponding cluster from NR group. [↑](#footnote-ref-1)
2. The proportion of this gene expressed in all cells of the corresponding cluster from HR group [↑](#footnote-ref-2)
3. The gene expression state in the corresponding cluster from NR group, “low” indicated that the expression level of corresponding genes in the NR group was lower than that in the HR group, “no” presented there was no significant difference in the expression of corresponding genes in NR group compared with HR group. [↑](#footnote-ref-3)
