# Supplementary material for: Single-cell RNA sequencing reveals the transcriptomic characteristics of peripheral blood mononuclear cells in hepatitis B vaccine non-responders
Source: Front Immunol. 2023 Aug 1;14:1091237. doi: 10.3389/fimmu.2023.1091237 (PMC10431960; doi:10.3389/fimmu.2023.1091237)
Supplement: Supplementary file 3 [file DataSheet_3.zip › Table 4.DOCX]

**Table 4. The number of DEGs of in naive B subset and memory B**

| **Group** | **Number of captured genes** | **Number of DEGs** | **X^2^** | **P** |
| --- | --- | --- | --- | --- |
| **Naive** | **3925** | **155** | **22.62** | **0.000** |
| **Memory** | **3479** | **72** |  |  |
