# Supplementary material for: Single-cell RNA sequencing reveals the transcriptomic characteristics of peripheral blood mononuclear cells in hepatitis B vaccine non-responders
Source: Front Immunol. 2023 Aug 1;14:1091237. doi: 10.3389/fimmu.2023.1091237 (PMC10431960; doi:10.3389/fimmu.2023.1091237)
Supplement: Supplementary file 3 [file DataSheet_3.zip › Table 5.DOCX]

| Cluster | Number of nodes | Number of edges | Average node degree | Avg. local clustering coefficient | Expected number of edges | PPI enrichment p-value |
| --- | --- | --- | --- | --- | --- | --- |
| Naive B | 141 | 436 | 6.18 | 0.467 | 208 | 1.00e-16 |
| Memory B | 68 | 83 | 2.44 | 0.479 | 48 | 4.12e-06 |

**Supplementary table 5. The PPI network information of DEGs**
