# Supplementary material for: Single-cell RNA sequencing reveals the transcriptomic characteristics of peripheral blood mononuclear cells in hepatitis B vaccine non-responders
Source: Front Immunol. 2023 Aug 1;14:1091237. doi: 10.3389/fimmu.2023.1091237 (PMC10431960; doi:10.3389/fimmu.2023.1091237)
Supplement: Supplementary file 3 [file DataSheet_3.zip › Table 6.DOCX]

**Supplementary table 6.** Parameter information for the MAPK related DEGs of Naive B and Memory B compared NR with HR group

| Cluster | Gene | P_val | avg_log2FC | pct.1^[[1]](#endnote-1)^ | pct.2^[[2]](#endnote-2)^ | P_val_adj | Change^[[3]](#endnote-3)^ |
| --- | --- | --- | --- | --- | --- | --- | --- |
| Naive B | FOS | 6E-21 | -0.976373552 | 0.39 | 0.638 | 7.95E-17 | low |
| Naive B | FOSB | 6E-20 | -0.71896823 | 0.23 | 0.462 | 8.02E-16 | low |
| Naive B | JUN | 3E-25 | -0.696909621 | 0.652 | 0.918 | 3.14E-21 | low |
| Naive B | JUNB | 6E-17 | -0.657151497 | 0.514 | 0.736 | 7.39E-13 | low |
| Naive B | JUND | 1E-14 | -0.420556564 | 0.779 | 0.975 | 1.62E-10 | low |
| Naive B | DDX5 | 4E-19 | -0.412853797 | 0.818 | 0.977 | 5.01E-15 | low |
| Naive B | DNAJA1 | 2E-08 | -0.311503049 | 0.229 | 0.375 | 2.12E-04 | low |
| Naive B | PDCD4 | 8E-07 | -0.273796354 | 0.392 | 0.546 | 1.02E-02 | low |
| Naive B | DUSP1 | 2E-23 | -0.67102004 | 0.472 | 0.75 | 2.12E-19 | low |
| Naive B | NFKBIA | 5E-15 | -0.550893023 | 0.422 | 0.641 | 6.08E-11 | low |
| Naive B | BLNK | 1E-08 | -0.345862422 | 0.193 | 0.33 | 1.78E-04 | low |
| Naive B | CXCR4 | 4E-12 | -0.412738937 | 0.765 | 0.981 | 5.21E-08 | low |
| Naive B | BLK | 3E-07 | -0.337554726 | 0.255 | 0.381 | 3.81E-03 | low |
| Naive B | HSPA8 | 4E-07 | -0.267442649 | 0.418 | 0.582 | 4.52E-03 | low |
| Naive B | UBA52 | 5E-17 | 0.277454737 | 0.998 | 0.994 | 6.49E-13 | high |
| Memory B | NFKBID | 2E-06 | -0.30459028 | 0.18 | 0.304 | 2.18E-02 | low |
| Memory B | JUN | 4E-13 | -0.517339366 | 0.679 | 0.876 | 4.44E-09 | low |
| Memory B | JUND | 4E-11 | -0.423401675 | 0.818 | 0.984 | 4.64E-07 | low |
| Memory B | DDX5 | 6E-12 | -0.33597678 | 0.872 | 0.993 | 7.60E-08 | low |
| Memory B | UBA52 | 1E-15 | 0.281180715 | 1 | 0.998 | 1.25E-11 | high |
| Memory B | DNAJA1 | 6E-12 | -0.381751307 | 0.304 | 0.498 | 8.11E-08 | low |
| Memory B | PDCD4 | 2E-07 | -0.282727282 | 0.447 | 0.621 | 2.04E-03 | low |
| Memory B | DUSP1 | 2E-11 | -0.414305082 | 0.544 | 0.77 | 1.96E-07 | low |

1. The proportion of this gene expressed in all cells of the corresponding cluster from NR group. [↑](#endnote-ref-1)
2. The proportion of this gene expressed in all cells of the corresponding cluster from HR group. [↑](#endnote-ref-2)
3. The gene expression state in the corresponding cluster from NR group compared with HR group, “low” indicated that the expression level of corresponding genes was lower, “high” indicated that the expression level of corresponding genes was higher, “no” presented there was no significant difference in the expression of corresponding genes. [↑](#endnote-ref-3)
