# Supplementary material for: Single-cell RNA sequencing reveals the transcriptomic characteristics of peripheral blood mononuclear cells in hepatitis B vaccine non-responders
Source: Front Immunol. 2023 Aug 1;14:1091237. doi: 10.3389/fimmu.2023.1091237 (PMC10431960; doi:10.3389/fimmu.2023.1091237)
Supplement: Supplementary file 3 [file DataSheet_3.zip › Table 7.DOCX]

**Supplementary table 7. The genes involved in the signaling pathway of interest**

| Cluster | biological processes | gene |
| --- | --- | --- |
| CD4^+^Teff | regulation of interleukin-4 production | HLA-DRB1/HLA-E/CEBPB/NDFIP1 |
| CD8^+^Teff | regulation of interleukin-4 production | HLA-DRB1/HLA-E/CEBPB |
| CD4^+^Teff | T cell receptor signaling pathway | TRBC1/HLA-DPA1/HLA-DRA/HLA-DPB1/HLA-DRB1/HLA-DRB5/TRAC/FYB1/HLA-A/PSMC5 |
| CD8^+^Teff | T cell receptor signaling pathway | TRBC1/HLA-DRB1/HLA-DPA1/HLA-DRA/HLA-DPB1/HLA-A/HLA-DRB5/PSMC5 |
| CD4^+^Teff | antigen receptor-mediated signaling pathway | TRBC1/HLA-DPA1/HLA-DRA/HLA-DPB1/HLA-DRB1/HLA-DRB5/TRAC/FYB1/HLA-A/PSMC5/PRKCB |
| CD8^+^Teff | antigen receptor-mediated signaling pathway | TRBC1/HLA-DRB1/HLA-DPA1/HLA-DRA/HLA-DPB1/HLA-A/HLA-DRB5/PSMC5 |
| CD4^+^Teff | T cell proliferation | HLA-DPA1/MSN/HLA-DPB1/HLA-DRB1/HLA-E/DOCK8/HMGB1/HLA-A/CEBPB/NDFIP1/ANXA1 |
| CD8^+^Teff | T cell proliferation | HLA-DRB1/HLA-DPA1/HLA-DPB1/HLA-A/RPS6/HLA-E/CEBPB |
